# Exposure to e-cigarette vapor extract induces vocal fold epithelial injury and triggers intense mucosal remodeling

**DOI:** 10.1242/dmm.049476

**Published:** 2022-08-23

**Authors:** Vlasta Lungova, Kristy Wendt, Susan L. Thibeault

**Affiliations:** 1 Department of Surgery, University of Wisconsin-Madison, 5105 WIMR Madison, WI 53705, USA; 2 Department of Surgery, University of Wisconsin-Madison, 5103 WIMR, Madison, WI 53705, USA

**Keywords:** Electronic cigarettes, Vaping, Chemical injury, Human vocal fold mucosa, Remodeling

## Abstract

Vaping has been reported to cause acute epiglottitis, a life-threatening airway obstruction induced by direct epithelial injury and subsequent inflammatory reaction. Here, we show that we were able to recapitulate this phenomenon *in vitro*. Exposure of human engineered vocal fold (VF) mucosae to 0.5% and 5% electronic cigarette (e-cigarette) vapor extract (ECVE) for 1 week induced cellular damage of luminal cells, disrupting homeostasis and innate immune responses. Epithelial erosion was likely caused by accumulation of solvents and lipid particles in the cytosol and intercellular spaces, which altered lipid metabolism and plasma membrane properties. Next, we investigated how the mucosal cells responded to the epithelial damage. We withdrew the ECVE from the experimental system and allowed VF mucosae to regenerate for 1, 3 and 7 days, which triggered intense epithelial remodeling. The epithelial changes included expansion of P63 (TP63)-positive basal cells and cytokeratin 14 (KRT14) and laminin subunit α-5 (LAMA5) deposition, which might lead to local basal cell hyperplasia, hyperkeratinization and basement membrane thickening. In summary, vaping presents a threat to VF mucosal health and airway protection, thereby raising further concerns over the safety of e-cigarette use.

This article has an associated First Person interview with the first author of the paper.

## Abstract (precis)

**Editor's choice:** Exposure of vocal fold mucosae to e-cigarette vapor extract induces lipid-mediated epithelial injury that disrupts vocal fold mucosal homeostasis and innate barrier function and triggers intense mucosal remodeling.

## INTRODUCTION

Recent cases of acute electronic cigarette (e-cigarette)- or vaping-associated lung injuries (EVALI) have opened a debate about the safety and health-related consequences of vaping. Multiple case reports have described atypical pneumonia and deaths in e-cigarette users (Ghinai et al., 2019; Layden et al., 2020; Davidson et al., 2019; Dicpinigaitis et al., 2020; Itoh et al., 2018), prompting intense scientific research focusing on the effects of vaping on distal airways, where gas exchange takes place (Blount et al., 2020; Madison et al., 2019; Chand et al., 2020). As e-cigarettes are heated in the mouth, inhaled vaporized e-cigarette liquids (e-liquids) pass through the throat, larynx and vocal folds (VFs) into the lungs. Droplet deposition in the upper airways can have physiological consequences and pose threats to oropharyngeal and VF health.

E-cigarettes are nicotine delivery systems that have been touted as safer alternatives to conventional smoking (Mirbolouk et al., 2018). E-cigarettes consist of prefilled or fillable cartridges that contain propylene glycol (PG) and vegetable glycerin (VG) (vehicle solvents), mixed with different concentrations of nicotine (N) and flavors (F). When heated in the mouth, e-liquids produce an aerosol that is inhaled and tends to adhere to exposed surfaces, such as soft and hard tissues (Sosnowski and Odziomek, 2018). Currently, an estimated 13 million people in the USA are active e-cigarette users (Mirbolouk et al., 2018). Those that have no previous experience with conventional cigarette smoking are at higher risk, as they exhibit increased susceptibility to lung damage and viral and/or bacterial infections (Gotts et al., 2019).

In this study, we evaluated the possible consequences of vaping on VF mucosal structure and function during e-cigarette vapor extract (ECVE) exposure and subsequent VF mucosal regeneration. The larynx and VFs are involved in voice production and are, as parts of the conducting airways, directly exposed to inhaled vaporized e-liquids. The VF epithelial cells are particularly vulnerable as they serve as the first line of defense against inorganic, organic and microbial intruders and protect the VF lamina propria beneath (Levendoski et al., 2014). Disruption in the VF epithelial barrier can cause acute laryngeal inflammation and swelling of laryngeal structures, which can rapidly lead to life-threatening airway obstruction. A recently reported case of epiglottitis in an adolescent female patient revealed a connection between vaping and swollen laryngeal structures, which seriously impaired voicing and breathing (Bozzella et al., 2020). The clinical course and biopsy findings showed signs of direct chemical epithelial injury, which led to subsequent inflammatory reaction (Bozzella et al., 2020). Despite the importance of understanding the biological effects of vaping on the laryngeal and VF mucosa, the physiological consequences of e-liquid deposition and whether this phenomenon can be recapitulated in experimental *in vitro* conditions remain unknown.

The recent development of a three-dimensional (3D) model of human VF mucosa by our group has allowed us to mimic *in vivo* remodeling of the VF mucosa in tobacco-related diseases (Lungova et al., 2019). Here, we demonstrate that exposure of engineered VF mucosae to 0.5% and 5% ECVE for 1 week induced cellular damage in VF luminal epithelial cells, disrupting mucosal homeostasis and mucosal innate immune responses. Epithelial erosion was likely caused by the accumulation of solvents and lipid particles, most likely medium chain fatty acids, in the cytosol and intercellular spaces, which altered lipid metabolism and plasma membrane properties. We next investigated how mucosal cells responded to the damage on the epithelial protective barrier. We withdrew the ECVE from the experimental system and allowed the VF mucosae to regenerate for 1, 3 and 7 days. Withdrawal of ECVE activated reparative processes in the VF epithelium, namely basal cell layer expansion, enhanced cytokeratin 14 (KRT14 or K14) and laminin subunit α-5 (LAMA5) deposition, which can lead to basal cell hyperplasia, hyperkeratinization and basement membrane thickening, which might alter VF mucosal properties.

Collectively, our experimental findings revealed that exposure of VF mucosae to vaporized e-liquids disrupts VF mucosal homeostasis and innate barrier functions. Further, reactive epithelial changes in response to this injury might represent consequential threat to VF mucosal health, thereby raising concerns over the safety of e-cigarette use for other vital and essential portions of the upper airway.

## RESULTS

### Histological alterations of VF mucosae exposed to 5% ECVE

**Fig. 1.**
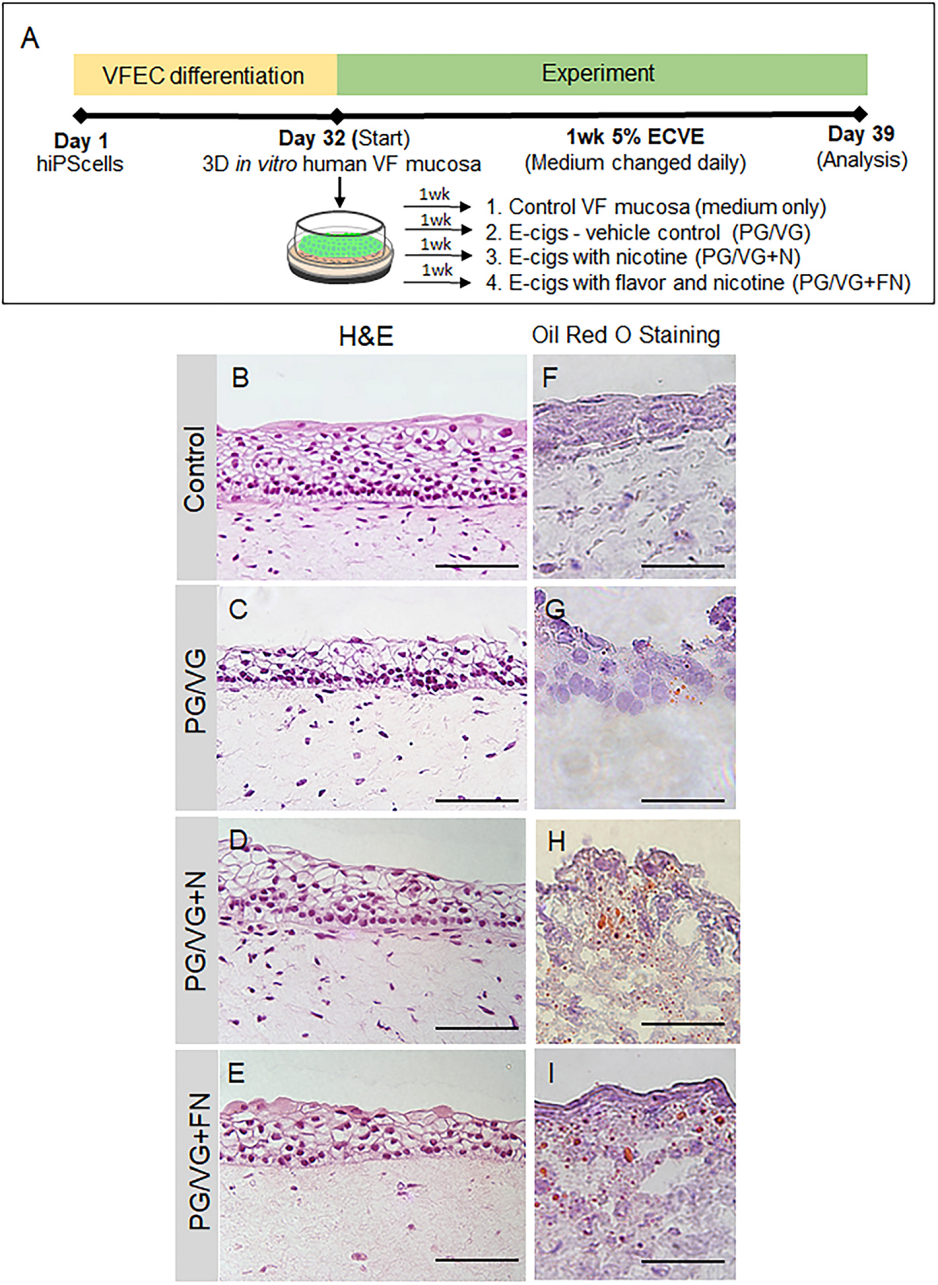
**Experimental design and morphology of hiPSC-derived VF mucosae exposed to 5% ECVE.** (A) Schematic illustration of the experimental design. HiPSCs were first differentiated into VF epithelial cells (VFECs) for 32 days and then exposed to 5% ECVE for 1 week. We tested three different conditions – e-cigarettes with PG/VG only, e-cigarettes with PG/VG and nicotine (PG/VG+N) and e-cigarettes with PG/VG with flavor and nicotine (PG/VG+FN). Control VF mucosae treated with plain culture medium were used as negative controls. At day 39, VF mucosae were collected and analyzed by immunohistochemistry and qPCR. (B-E) Morphology of VF mucosae in a control group (B) and 5% ECVE-exposed groups (C-E) showing stratified squamous VF epithelia. (F-I) Oil Red O staining of frozen unfixed sections of VF mucosae in the control group (F) and 5% ECVE-exposed groups (G-I). All 5% ECVE treated samples contained lipid droplets that adhered to cell surfaces. Scale bars: 100 µm (B-E) and 50 µm (F-I). The histology datasets were performed in three biological and two technical replicates (*n*=3) and were repeated twice in the laboratory by two investigators.

To investigate the effect of ECVE on human VF mucosal homeostasis, we utilized the recently developed human induced pluripotent stem cell (hiPSC)-derived model of human VF mucosae (Lungova et al., 2019). HiPSCs were first differentiated into VF basal epithelial progenitors for 10 days, reseeded on collagen-fibroblast constructs and were allowed to differentiate for 22 days, first as submerged cultures and then at the air/liquid interface (A/Li). At day 32, engineered VF mucosae were exposed to 5% ECVE for 1 week to mimic exposure of cells to vaping (Fig. 1A) (for the detailed protocol, see the Materials and Methods). VF mucosae treated with plain culture medium were used as negative controls. For experimental groups, we tested three different types of e-cigarette vapor extracts including vehicle controls with polypropylene glycol and vegetable glycerin (PG/VG) only, e-cigarettes with PG/VG and nicotine (PG/VG+N) and e-cigarettes with PG/VG with flavor and nicotine (PG/VG+FN), the most popular types of e-cigarettes. At day 39, human engineered VF mucosae were collected for analysis.

First, we investigated alterations in the morphology of VF mucosae in control and experimental groups. Histological assessment of the VF mucosae showed a typical stratified squamous architecture for all conditions (Fig. 1B-E). Epithelial cells were arranged in a basal cell layer and suprabasal cell layers that flattened apically (Fig. 1B-E). Overall histological examination revealed that ECVE-exposed VF mucosae appeared thinner than the controls (Fig. 1B-E). Next, we performed Oil Red O staining on frozen unfixed sections and found red oil droplets on the surface of the VF epithelium in all ECVE-exposed VF mucosae (Fig. 1F-I), suggesting that the e-liquid contained lipid components. When heated, the e-liquid produced aerosols that mixed with the culture medium and deposited lipid droplets that adhered to cell surfaces. In order to examine whether the exposure of cells to ECVE could alter the structure and function of the VF epithelial barrier, we evaluated the expression of stratified epithelial markers and tested the functionality of VF epithelial cells by assessing mucin and inflammatory cytokine/chemokine expression.

### ECVE exposure affects compactness of apical epithelial cell layers

We have previously demonstrated that exposure of engineered VF mucosae to 5% cigarette smoke extract (CSE) leads to VF mucosa remodeling, predominantly affecting the basal epithelial cell layer with downregulation of K14, which pathologically accumulates in the luminal cells along with cytokeratin 13 (KRT13 or K13) (Lungova et al., 2019). Therefore, we sought to determine whether 1 week exposure to 5% ECVE could also affect cytokeratin production and the structure of the basal cellular compartment. We first evaluated K14 and LAMA5 expression and co-stained with P63 (encoded by *TP63*), a marker of basal cells. We found that K14 staining was strong in 5% ECVE-treated groups and controls (Fig. S1A-D). Similarly, LAMA5, a marker of the basement membrane, was detected in both control and 5% ECVE-treated groups (Fig. S1E-H) along with P63 (Fig. S1A-H). However, there was a reduced expression of suprabasal K13 in ECVE-exposed groups, as compared to that seen in controls, suggesting that apical epithelial surfaces are compromised by ECVE exposure (Fig. S1I-L). To assess the compactness of the epithelial barrier, we stained for E-cadherin (ECad, encoded by *CDH1*), a marker of cell adherent junctions. We found that in control samples, ECad was strongly expressed in all epithelial cell layers (Fig. S1M), whereas in 5% ECVE-exposed groups, the ECad signal was absent in some cells in the apical cell layers (Fig. S1N-P). Histological data were supported by quantitative PCR (qPCR) (Fig. S1Q-T). As expected, the transcript levels of *K14* were upregulated in the PG/VG and PG/VG+N groups and did not change in the PG/VG+FN group as compared to the control (Fig. S1Q). *TP63* transcript levels remained the same in PG/VG and PG/VG+N groups compared to the controls and significantly decreased in the PG/VG+FN group (Fig. S1R). On the other hand, unlike in the control group, the expression levels of *K13* were significantly reduced in ECVE-exposed groups (Fig. S1S). For ECad, we found a significant downregulation of *CDH1* in the PG/VG+FN group only, compared to the controls or other experimental groups (Fig. S1T). These data indicate that exposure of the VF epithelium to 5% ECVE likely impairs the structure and integrity of the luminal epithelial cell layers that come into direct contact with aerosol and toxic substances found in ECVE. Moreover, the decreased expression of *TP63* in the PG/VG+FN group suggests that the damage to the luminal layers can also ultimately cause changes in basal cells.

### ECVE exposure alters the expression of a membrane-associated mucin

We further investigated whether exposure to 5% ECVE can alter the function of the VF epithelial protective barrier. We have previously shown that the VF epithelium is an essential mechanism of VF defense (Levendoski et al., 2014), which is achieved by the compact physical epithelial barrier, mucus production and secretion of cytokines/chemokines that are responsible for innate immunity. Above, we showed that 5% ECVE exposure affected cell adherent junctions and K13 expression in the luminal cell layers. Next, we evaluated the expression of mucins 1 (MUC1) and 4 (MUC4), which are typical membrane-associated mucins present in the human VFs (Levendoski et al., 2014; Lungova et al., 2019). Secretory proteins, such as MUC5B or MUC5AC, are not produced by stratified VF epithelial cells that cover the membranous portion of the true VFs (Levendoski et al., 2014). Our histological data confirmed that in control VF mucosae, MUC1 was detected in the apical cell layer and formed a thin protective coat (Fig. S2A), as previously shown in human native VF mucosae (Lungova et al., 2019). In the 5% ECVE-treated groups PG/VG and PG/VG+N, MUC1 expression was upregulated and was observed in deeper epithelial cell layers (Fig. S2B,C). Notably, in the PG/VG+FN group, the MUC1 layer remained thin, but we observed mucus clots on the epithelial surface that wrapped cell debris (Fig. S2D). As for MUC4, we did not observe any significant changes in the expression patterns between control and ECVE-exposed groups (Fig. S2E-H). We further confirmed the expression of mucins by qPCR. In the PG/VG and PG/VG+N groups, *MUC1* transcript levels were upregulated, whereas in the PG/VG+FN group, the expression levels of *MUC1* were similar to those in the control group (Fig. S2I). On the other hand, transcript levels of *MUC4* decreased significantly in VF mucosae exposed to PG/VG+N and PG/VG+FN (Fig. S2I). These findings show that exposure to ECVE leads to increased expression of luminal *MUC1*, but not of *MUC4*, and to the formation of mucus clots that can accumulate in the laryngeal/airway lumen and impair mucus clearance. Next, we assessed whether structural and functional changes in the VF epithelium were capable of inducing the expression of cytokines and activate a VF mucosal immune response.

### ECVE exposure stimulates the production of chemokines implicated in the recruitment of eosinophils

To better understand the immunomodulatory consequences of ECVE exposure, we evaluated VF mucosal cytokine and chemokine profiles for control versus ECVE-exposed VF mucosae. We performed SYBR Green-based quantitative real-time RT^2^ PCR profiling array looking at genes involved in human cytokine and chemokine expression. We used 384-well profiler plates that were designed to screen all four samples on one plate (a 4×96-well format). Samples were run in triplicate (*n*=3; 12 samples total). For each sample, the 384-well plate contained primers for 84 genes involved in human cytokine and chemokine expression, five housekeeping genes and three negative control wells (Table S1). Cycle threshold (CT) values, fold-regulation values and *P*-values for all tested genes are given in Tables S2 and S3. Gene expression levels were normalized to reference genes and control samples. Positive fold-regulation values indicate upregulated genes, whereas negative values indicate downregulated genes (Table S3). Genes with a fold change ≥2 and *P*≤0.05 were considered as significantly differentially expressed genes.

**Fig. 2.**
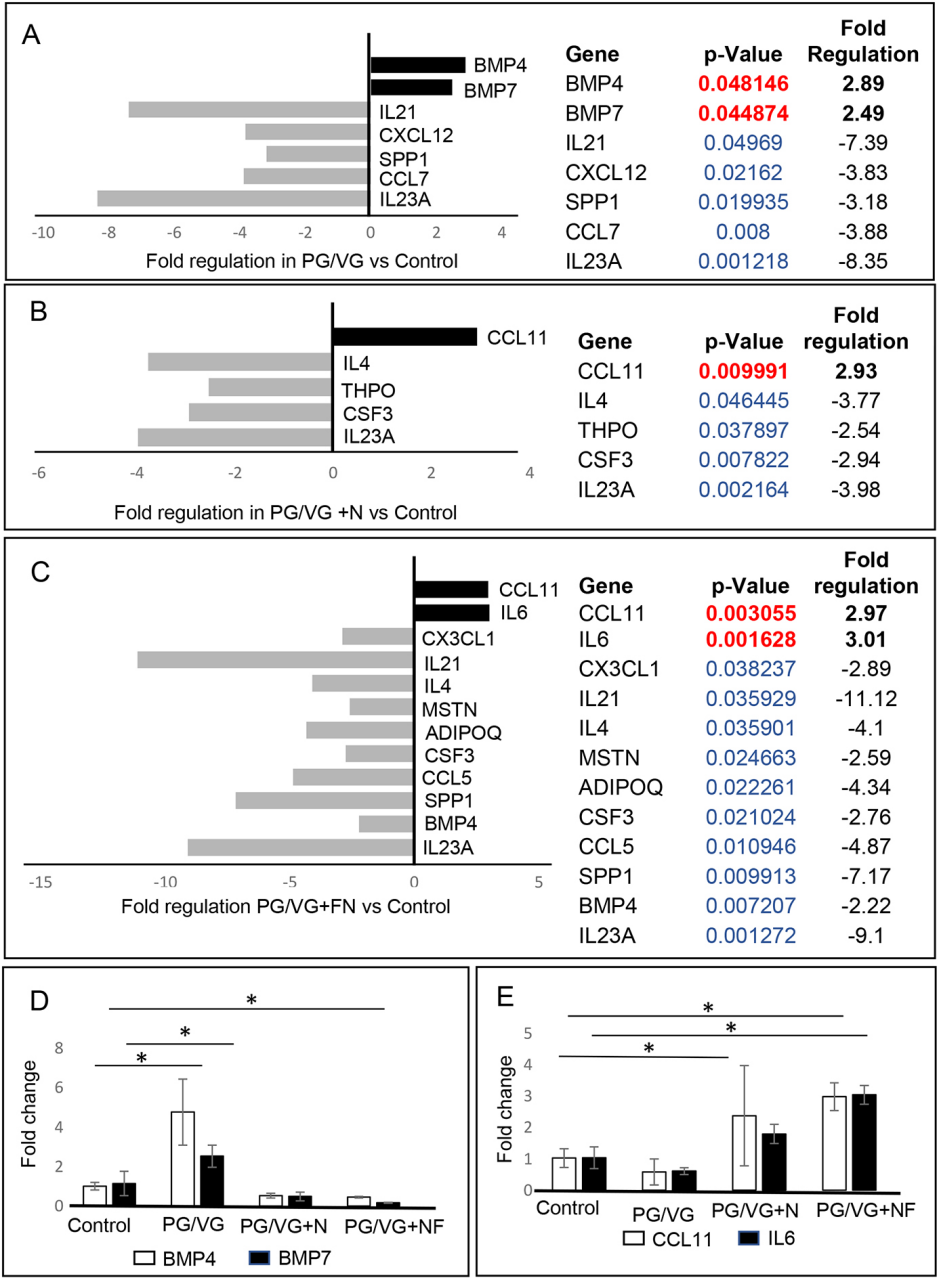
**RT^2^ PCR profiling analysis focusing on human cytokine and chemokine expression.** (A-C) Significantly differentially expressed genes involved in mucosal inflammation in PG/VG versus control (A), PG/VG and nicotine versus control (B) and PG/VG and flavor and nicotine versus control (C). Upregulated genes with positive fold-regulation values are highlighted in black. Downregulated genes with negative fold-regulation values are highlighted in gray. Fold-change regulations and statistical analyses were calculated using the web portal at http://www.qiagen.com/geneglobe and are presented as column graphs without error bars (error bar calculations were not included in the final reports). The fold-change threshold was set to 2. *P-*values were calculated based on a two-tailed unpaired Student's *t*-test of the replicate 2^−ΔΔCT^ values for each gene in the control and treatment groups and *P*<0.05 was considered as significant. *P*-value calculation used was based on parametric, unpaired, two-sample equal variance and two-tailed distribution. Samples were run in triplicate (*n*=3, 12 samples total). (D,E) Overlap in expression levels of *BMP4* and *BMP7* (D) and *CCL11* and *IL6* (E) in control and experimental groups. The CT values were manually pulled from the datasets and fold-change regulation was calculated using 2^−ΔΔCT^. Error bars represent the mean±s.e.m. from three biological replicates (*n*=3). One-way ANOVA with Tukey’s HSD test were used to confirm statistical significance in gene expression; *P≤0.05.

Our results revealed that exposure of the VF mucosae to 5% ECVE dysregulated inflammatory responses in epithelial cells and VF fibroblasts (Fig. 2). In the PG/VG group versus the control group, we found significant modulation in seven genes (Fig. 2A), with two upregulated genes [*BMP4* (+2.89-fold) and *BMP7* (+2.49-fold)] and five downregulated genes [*SPP1* (−3.18-fold), *IL23A* (−8.35-fold), *IL21* (−7.39-fold), *CXCL12* (−3.83-fold) and *CCL7* (−3.88-fold)]. In Fig. 2D, we show the overlap in the expression of *BMP4* and *BMP7* across all time points in control and experimental groups, highlighting the significant upregulation of these genes in the PG/VG group compared to the PG/VG+N and PG/VG+FN groups. These findings suggest that nicotine might suppress BMP signaling in VF mucosal cells.

In the PG/VG+N group, we identified five genes with expression levels that were significantly different from those in the control group (Fig. 2B), with one upregulated gene [*CCL11* (+2.93-fold)] and four suppressed genes [*THPO* (−2.54-fold), *IL4* (−3.77-fold), *IL23A* (−3.98-fold) and *CSF3* (−2.94-fold)]. When we compared the PG/VG+FN group to the control group, 12 genes showed significant differential expression (Fig. 2C); *IL6* (+3.01-fold) and *CCL11* (+2.97-fold) were upregulated and *SPP1* (−7.17-fold), *MSTN* (−2.59-fold), *IL4* (−4.10-fold), *IL23A* (−9.10-fold), *IL21* (−11.12-fold), *CX3CL1* (−2.89-fold), *CSF3* (−2.76-fold), *CCL5* (−4.87-fold), *BMP4* (−2.22-fold) and *ADIPOQ* (−4.34-fold) were downregulated. These data suggest that the PG/VG+FN treatment dysregulates VF mucosal cytokine production to a greater extent than PG/VG and PG/VG+N treatment. Suppression of chemokines, such as *CCL5*, *CCL7*, *CX3CL1*, *IL23A*, *IL21* and *CSF3*, might delay mucosal VF response to pathogens (Madison et al., 2019), as these chemokines are implicated in the recruitment of monocytes/macrophages and neutrophils to the site of inflammation (Griffith et al., 2014; Lim et al., 2020). Suppression of cytokines with anti-inflammatory cytoprotective function, such as *IL4* (Russell and Morgan, 2014), might delay VF mucosal repair and regeneration. On the other hand, consistently elevated transcript levels of *CCL11* and *IL6* in the PG/VG+N and PG/VG+FN groups (Fig. 2E) demonstrate that nicotine can activate the secretion of proinflammatory cytokines that stimulate the migration of eosinophils, which are otherwise involved in allergic reactions (Glaser et al., 2019), and induce moderate mucosal inflammation.

### ECVE exposure causes imbalance in cytosolic lipid content and alters plasma membrane properties

**Fig. 3.**
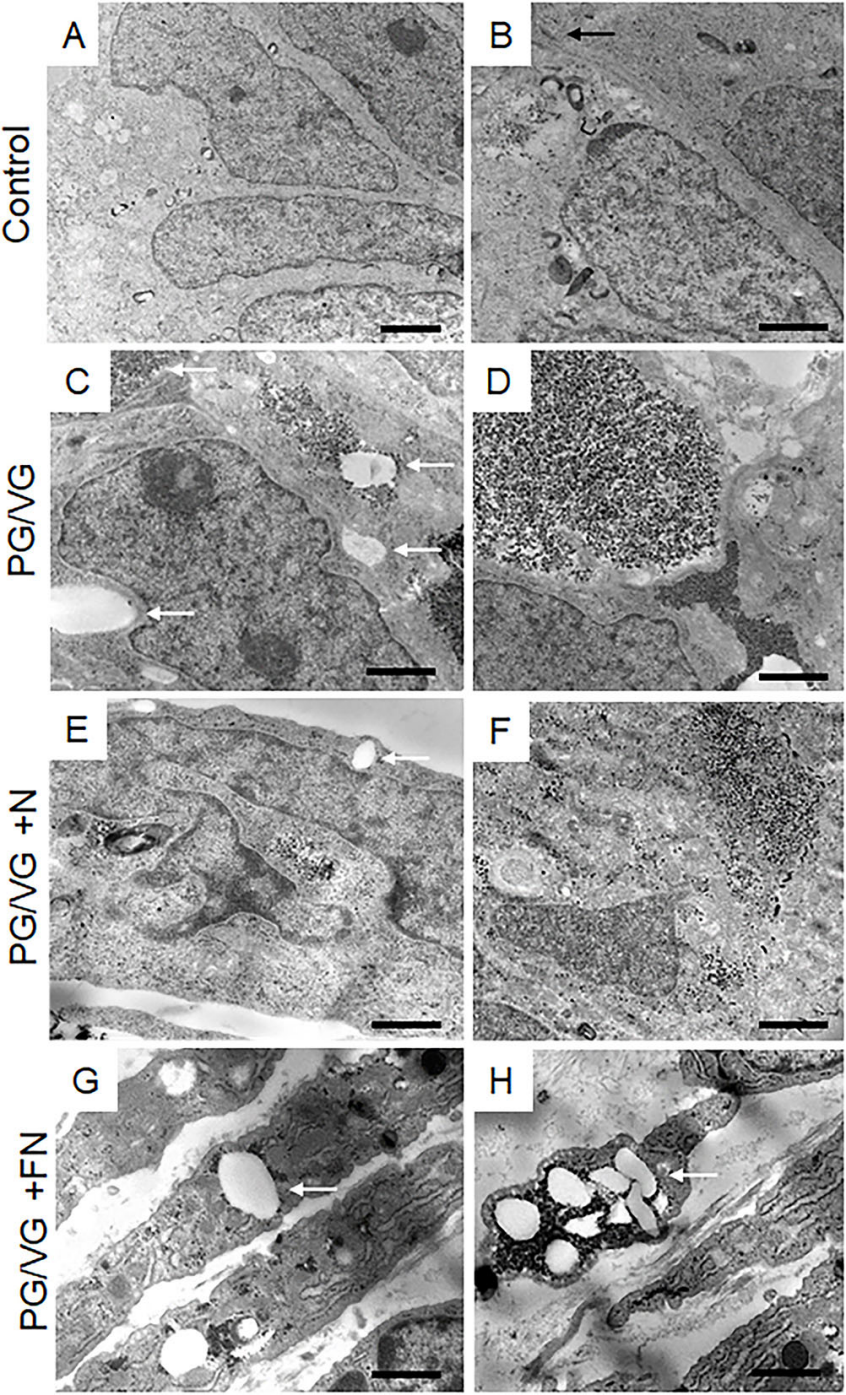
**Transmission electron microscopy of the apical region of the VF epithelium.** (A,B) Ultrastructure of VF epithelial cells of control VF mucosae. The black arrow indicates the tight junction in a control group. (C,D) Ultrastructure of VF epithelial cells exposed to PG/VG only. The cytosol of the epithelial cells contains dark droplets and/or pigments. White solid arrows denote white aggregates that accumulate in the cytosol and intercellular space. (E,F) Ultrastructure of VF epithelial cells exposed to PG/VG and nicotine. White arrows point to white lipid aggregates in the cell cytoplasm. Apical cells tend to detach from the underlying epithelial cell layers. (G,H) Ultrastructure of VF epithelial cells exposed to PG/VG and nicotine and flavor. White solid arrows point to white lipid aggregates and dark droplets in the cytoplasm of detached cells. Some exfoliated cells became loaded with lipid particles and dark pigments and resembled lipid laden macrophages. Scale bars: 800 nm (A,C,E,G,H); 200 nm (B,D,F).

We further investigated whether altered VF epithelial structure and function were associated with oil droplet deposition and examined the ultrastructure of VF epithelial cells with transmission electron microscopy (TEM). In ECVE-exposed VF mucosae, but not in controls, we found numerous tiny dark spots (likely droplets) along with large white lipid aggregates inside the cytoplasm and intercellular spaces (Fig. 3A-H). The presence of inclusions between plasma membranes dilated the space between neighboring cells and impaired cell junctions (Fig. 3C). Moreover, in the PG/VG+N and PG/VG+FN groups, the outermost epithelial cells were detached from the underlying cell layers (Fig. 3E,G) and some exfoliated cells were loaded with lipid particles and dark pigments (Fig. 3G,H) and resembled lipid laden macrophages (Blount et al., 2020; Madison et al., 2019). The origin of the cytoplasmic lipid aggregates and/or dark pigments is not clear. As VF epithelial cells are not active producers of lipid components, such as pulmonary surfactants (Madison et al., 2019), we speculate that these are from exogenous sources. Even though VF epithelial cells are not active lipid producers, they possess the enzymatic machinery involved in lipid/fatty acid metabolism to maintain a phospholipid bilayer and vital cellular functions. To investigate whether the cytoplasmic accumulation of lipid droplets could affect VF mucosal cell metabolic activity related to fatty acid/glycerol breakdown and/or recycling, we performed SYBR Green-based quantitative real-time RT^2^ PCR profiling, looking at genes involved in human fatty acid metabolism. As described above, a 4×96-well format (384-well plates) was used to screen all four samples on one plate and data were run on three separate plates (*n*=3, 12 samples total). For each sample, the 384-well plate contained primers for 84 genes involved in human fatty acid metabolism, five housekeeping genes and three negative control wells (Table S4). CT values, fold-regulation values and *P*-values for all tested genes are included in Tables S5 and S6. As mentioned above, positive fold-regulation values indicate upregulated genes, whereas negative fold-regulation values indicate downregulated genes (Table S6). Genes with a fold change ≥2 and *P*≤0.05 were considered as significantly differentially expressed genes.

**Fig. 4.**
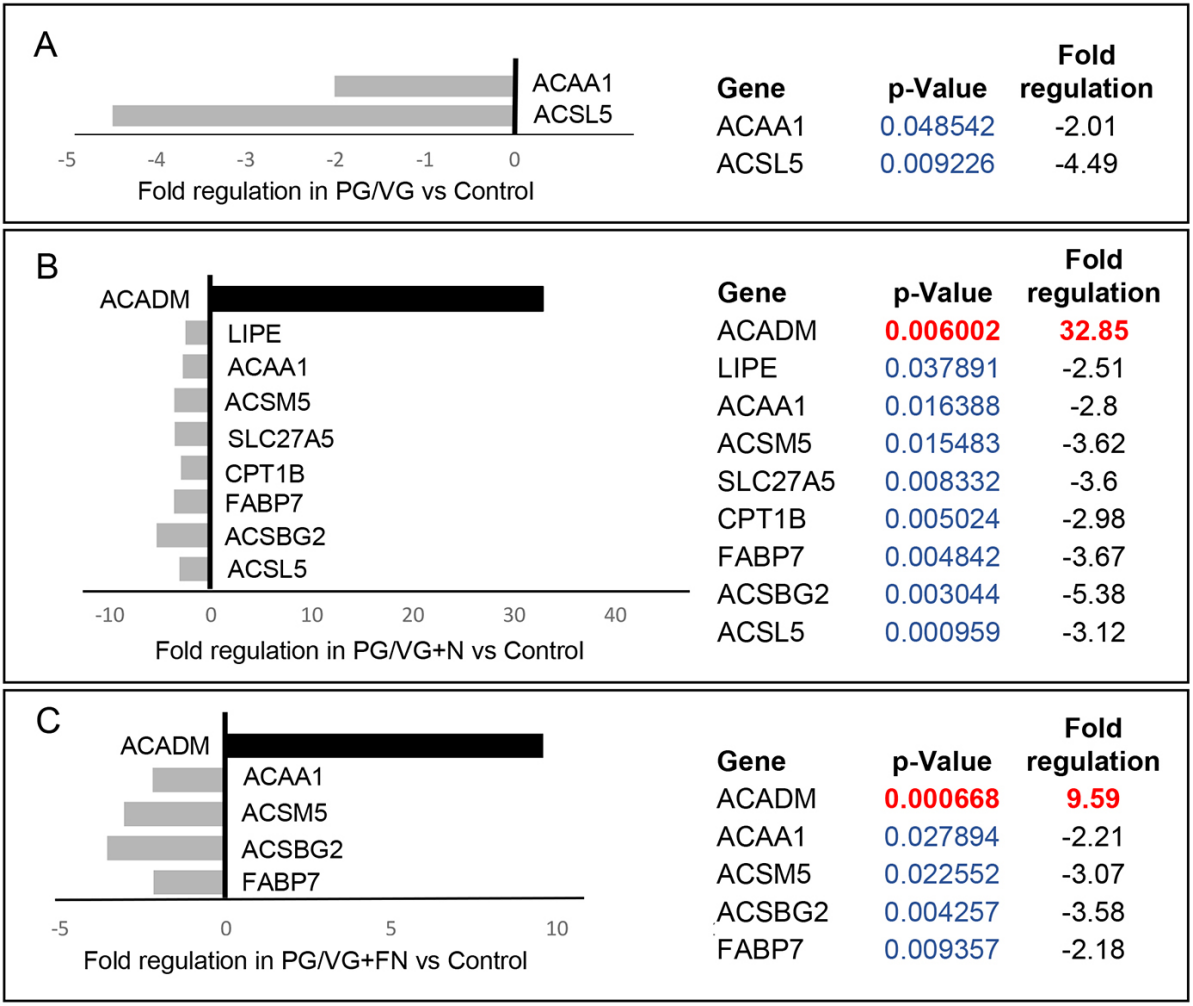
**RT^2^ PCR profiling analysis focusing on human fatty acid metabolism.** (A-C) Significantly differentially expressed genes involved in fatty acid metabolism in PG/VG versus control (A), PG/VG and nicotine versus control (B) and PG/VG and flavor and nicotine versus control (C). Upregulated genes with positive fold-regulation values are highlighted in black. Downregulated genes with negative fold-regulation values are highlighted in gray. Fold-change regulations and statistical analyses were calculated using the web portal at http://www.qiagen.com/geneglobe and are presented as column graphs without error bars (error bar calculations were not included in the final reports). The fold-change threshold was set to 2. *P-*values were calculated based on a two-tailed unpaired Student's *t*-test of the replicate 2^−ΔΔCT^ values for each gene in the control and treatment groups and *P*<0.05 was considered as significant. *P*-value calculation used was based on parametric, unpaired, two-sample equal variance and two-tailed distribution. Samples were run in triplicate (*n*=3, 12 samples total).

Our results show that the PG/VG group was at least affected by 5% ECVE as only two genes were significantly different from controls. *ACAA1* (−2.01-fold) and *ACSL5* (−4.49-fold) were significantly downregulated (Fig. 4A). In other experimental groups, i.e. PG/VG+N and PG/VG+FN, the effect of ECVE treatment was more obvious (Fig. 4B,C). We found nine and five significantly differentially expressed genes in the PG/VG+N and PG/VG+FN groups, respectively. Upregulated genes included *ACADM* with a fold change of 32.85 in the PG/VG+N group and 9.59 in the PG/VG+FN group. This gene encodes the enzyme, acyl-coenzyme A dehydrogenase, which is involved in medium-chain fatty acid degradation in mitochondria (Jensen et al., 2012). On the other hand, the downregulated genes [*ACSBG2* (−5.38-fold), *ACSL5* (−3.12-fold), *ACSM5* (−3.62-fold) and *CPT1B* (−2.98-fold) in the PG/VG+N group and *ACSBG2* (−3.58-fold) and *ACSM5* (−3.07-fold) in the PG/VG+FN group] encode enzymes needed for the synthetic reaction of fatty acids with acyl-coenzyme A and adenosine triphosphate in the cytosol, which is required for fatty acid activation and their translocation into mitochondria (Grevengoed et al., 2014; Schönfeld and Wojtczak, 2016). Notably, the significantly downregulated gene *ACSM5* in the PG/VG+N and PG/VG+FN groups is also involved in medium-chain triglyceride/fatty acid degradation (Schönfeld and Wojtczak, 2016). Moreover, we found a significant downregulation of fatty acid-binding protein *FABP7*, with −3.67-fold expression in the PG/VG+N group and −2.18-fold expression in the PG/VG+FN group, along with *SLC27A5*, with −3.60-fold expression in the PG/VG+N group. The proteins expressed by these genes facilitate the transport of fatty acids/triglycerides across the plasma membrane and within the cytoplasm and, thus, regulate lipid cytoplasmic content (Schwenk et al., 2010).

Overall, these findings show that the inefficient clearance of lipids and solvents in the cytosol dysregulates lipid metabolism and plasma membrane properties and causes chemical VF epithelial injury. We then sought to further elucidate the consequences of ECVE exposure on VF mucosal structure and function and the potential mechanisms that control epithelial remodeling.

### Lipid-mediated VF injury triggers epithelial remodeling

**Fig. 5.**
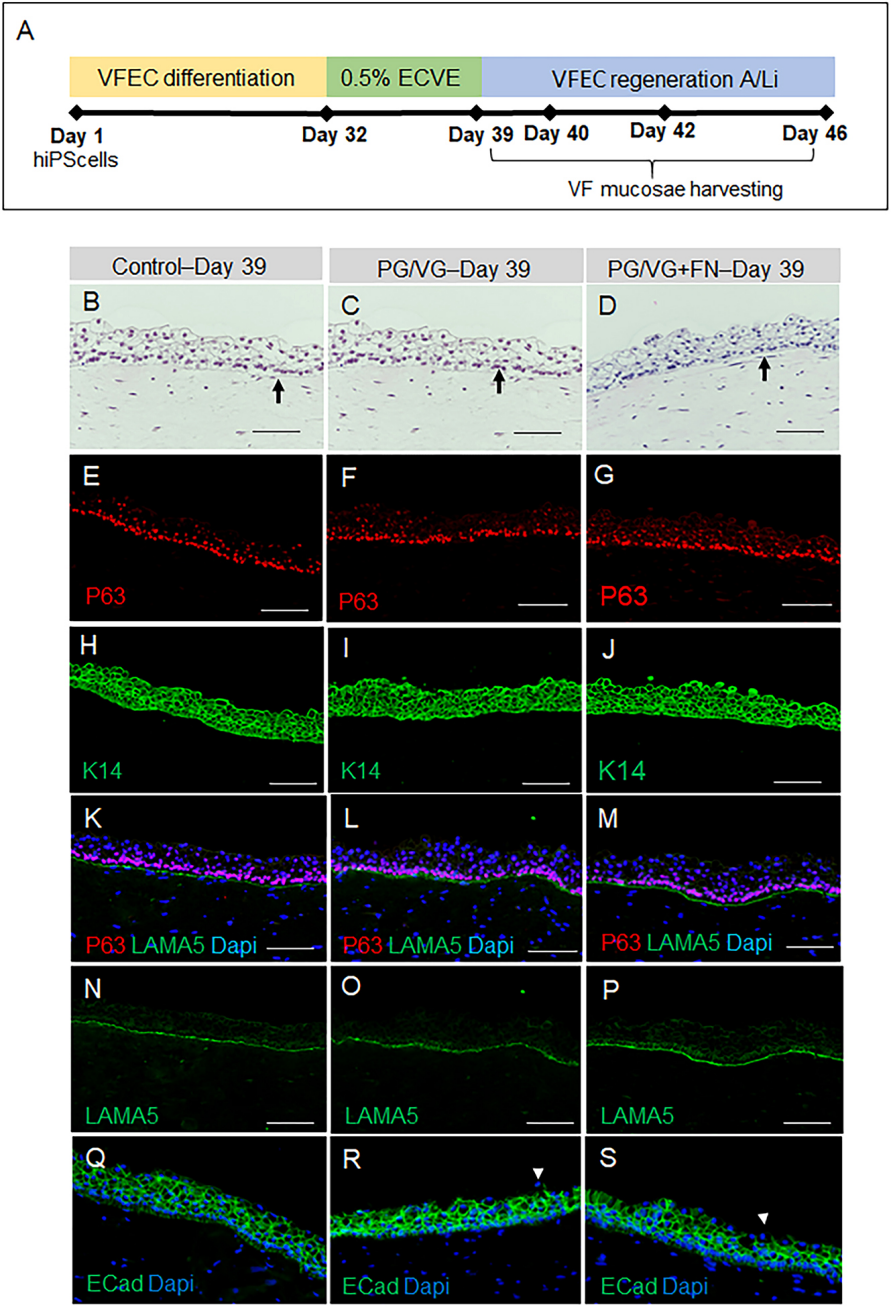
**VF epithelial recovery and morphology of hiPSC-derived VF mucosae exposed to 0.5% ECVE at day 39.** (A) Schematic illustration of the experimental design for assessment of VF epithelial recovery. HiPSCs were first differentiated into VF epithelial cells (VFECs) for 32 days and then exposed to 0.5% ECVE for 1 week. At day 39 (0 days post exposure), ECVE was withdrawn from the experimental system and VF mucosae were allowed to regenerate for 1 day (day 40), 3 days (day 42) and 7 days (day 46) in regular FAD medium at the A/Li. At selected time points, VF mucosae were harvested and analyzed. (B-D) Morphology of VF mucosae in the control group (B) and 0.5% ECVE-exposed groups (C,D) showing stratified squamous VF epithelia. Black arrows denote the basal cellular compartment. (E-G) Anti-P63 staining (red) in control (E) and 0.5% ECVE-exposed VF mucosae (F,G). (H-J) Anti-cytokeratin 14 staining (green) in control (H) and 0.5% ECVE-exposed VF mucosae (I,J). (K-M) Anti-laminin subunit α-5 (green) co-stained with anti-P63 (red) in control (K) and 0.5% ECVE-treated VF mucosae (L,M). (N-P) Anti-laminin subunit α-5 as a single green channel in control (N) and 0.5% ECVE-exposed VF mucosae (O,P). (Q-S) Anti-E-cadherin staining (in green) in control (Q) and 0.5% ECVE-exposed VF mucosae (R,S). White arrowheads in panels R and S point to apical cells with decreased E-cadherin expression. The histology datasets were performed in three biological and two technical replicates (*n*=3) and were repeated twice in the laboratory by two investigators. Scale bars: 100 µm.

To investigate how mucosal cells respond to damage to the epithelial protective barrier, we first created engineered VF mucosae and induced VF epithelial injury at day 32 by exposing cells to 0.5% ECVE for 1 week (Fig. 5A). We decreased ECVE concentration from 5% to 0.5% to reduce cell damage and allow cells to recover in *in vitro* conditions that can otherwise be challenging owing to the absence of supporting immune cells and/or nutrients from the bloodstream. We included PG/VG (vehicle control) and PG/VG+FN in the experiment; VF mucosae treated with plain culture medium were used as negative controls. After 1 week of 0.5% ECVE exposure (day 39), we withdrew the ECVE from the experimental system and allowed the VF mucosae to regenerate for 1, 3 and 7 days at the A/Li (Fig. 5A). These time points were determined by previous studies using animal models that focused on VF mucosal tissue repair in response to VF injury and phonotrauma (Yamashita et al., 2009; Leydon et al., 2014; Rousseau et al., 2017; Yamashita et al., 2010). These studies have shown that the first 3 days post injury are associated with the acute phase of VF mucosal healing and cell proliferation, whereas the later time points (from day 3 to 7 or 14) refer to the remodeling phase characterized by increased cytokeratin and extracellular matrix deposition. At the selected time points, VF mucosae were harvested and analyzed.

We assessed the epithelial morphology, the structure of the basal cellular compartment by staining for P63, K14 and LAMA5, and the integrity of the epithelial barrier by staining for ECad. Overall, VF mucosal morphology was similar in both control and experimental groups at day 39 (day 0 post ECVE exposure) (Fig. 5B-D). All VF mucosae had a defined basal layer of P63^+^ cells (Fig. 5E-G) and basal cells expressed K14, which diffused into the suprabasal cell layers (Fig. 5H-J). Control and ECVE-exposed VF mucosae expressed LAMA5 in the basement membrane that co-stained with P63 (Fig. 5K-P). Our results also show that epithelial integrity was maintained, as we detected robust ECad staining throughout the entire VF epithelium in the control group (Fig. 5Q) and in experimental groups, with a few luminal cells that showed reduced ECad expression (Fig. 5R,S). These findings suggest that 0.5% ECVE caused milder epithelial injury than 5% ECVE as described above.

**Fig. 6.**
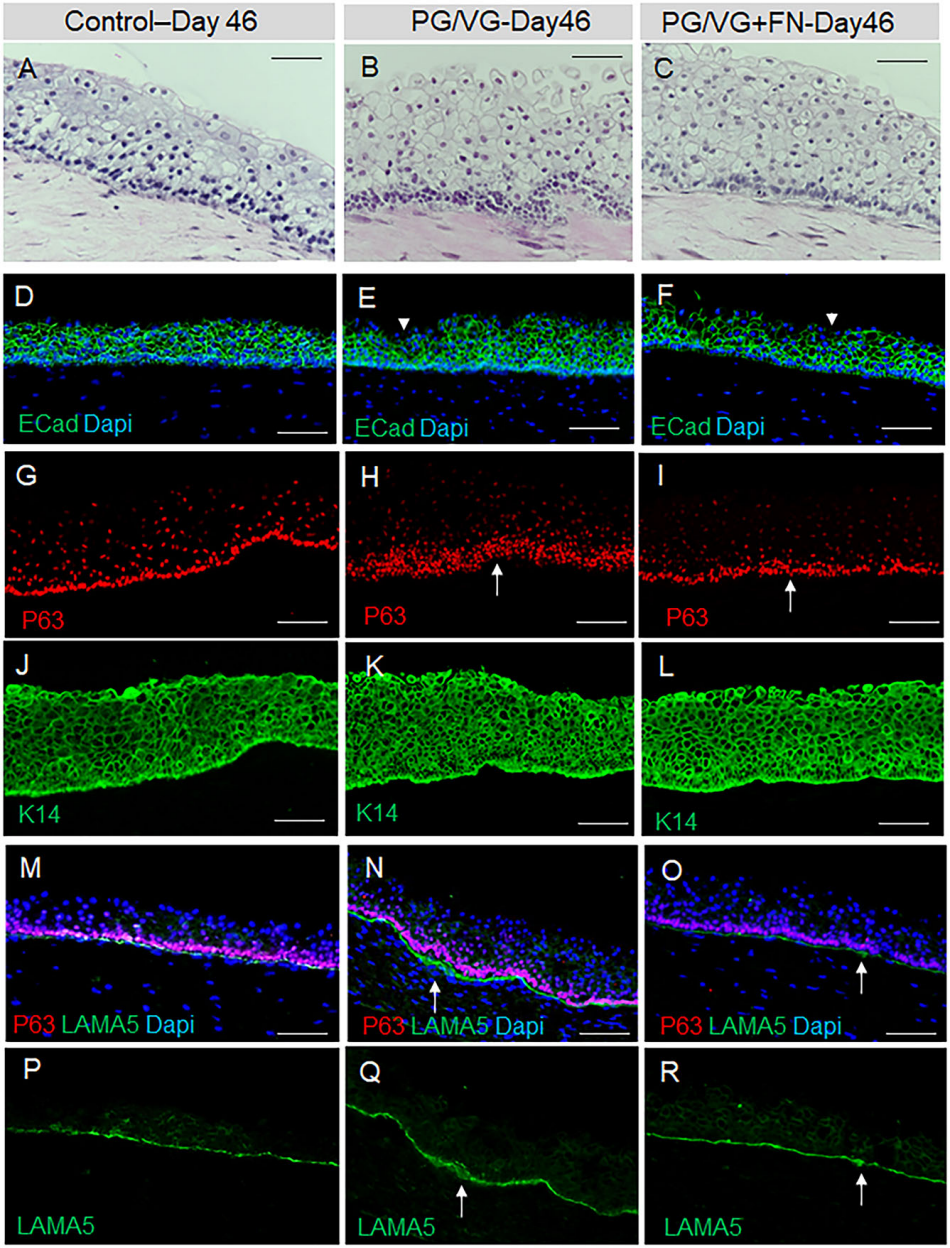
**HiPSC-derived VF mucosae exposed to 0.5% ECVE at day 46.** (A-C) Morphology of VF mucosae in control group (A) and 0.5% ECVE-exposed groups (B,C) at day 46 (7 days post exposure) showing thickened stratified squamous VF epithelia and tightly packed cells in the basal cellular compartment, particularly in the PG/VG group (B). (D-F) Anti-E-cadherin staining (in green) in control (D) and 0.5% ECVE-exposed VF mucosae (E,F). White arrowheads in panels E and F point to apical cells with decreased expression of E-cadherin. (G-I) Anti-P63 staining (red) in control (G) and 0.5% ECVE-exposed VF mucosae (H,I). White arrows in panels H and I denote tightly packed P63^+^ cells indicating epithelial hyperplasia. (J-L) Anti-cytokeratin 14 staining (green) in control (J) and 0.5% ECVE-exposed VF mucosae (K,L). (M-O) Anti-laminin subunit α-5 (green) co-stained with anti-P63 (red) in control (M) and 0.5% ECVE-treated VF mucosae (N,O). (P-R) Anti-laminin subunit α-5 as a single green channel in control (P) and 0.5% ECVE-exposed VF mucosae (Q,R). White arrows in panels N,O,Q,R denote local thickening of the basement membrane. The histology datasets were performed in three biological and two technical replicates (*n*=3) and were repeated twice in the laboratory by two investigators. Scale bars: 100 µm.

**Fig. 7.**
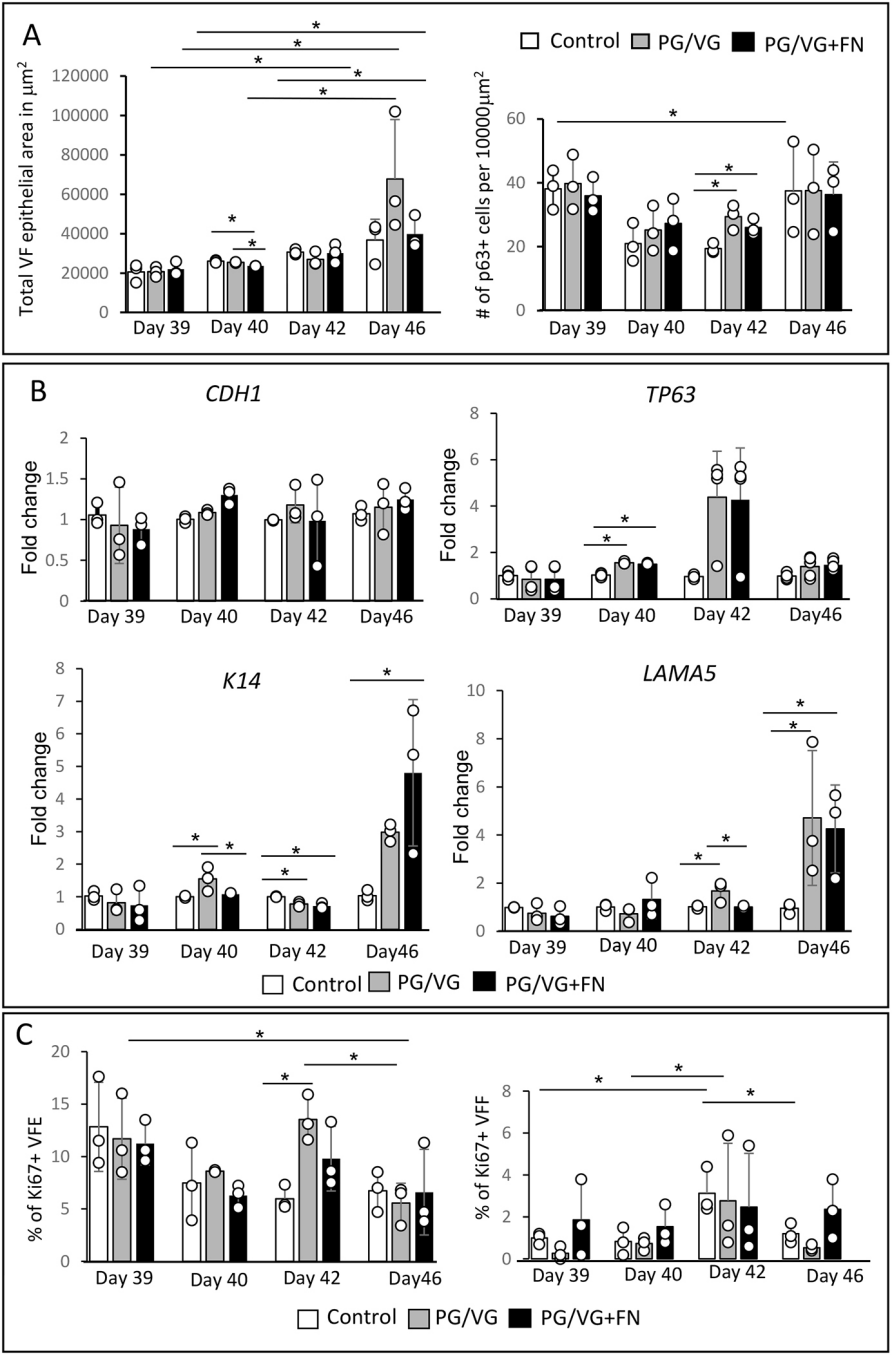
**Quantitative analyses and transcript levels of genes expressing VF epithelial structural proteins after 0.5% ECVE exposure during VF epithelial regeneration.** (A) Total VF epithelial area in µm^2^ and P63^+^ basal cell density calculated as the number of P63^+^ cells per 10,000 µm^2^ in control, PG/VG and PG/VG+FN groups at different stages of epithelial recovery. All calculations were performed as blinded experiments and calculated independently by two researchers. Data presented were obtained from three biological replicates (*n*=3), ten images were included in the analyses for each control and experimental condition, time point and biological replicate (360 images in total). Error bars with individual data points (average values for each condition) represent ±s.e.m. of the mean. (B) Transcript levels of the genes expressing VF epithelial structural proteins, i.e. *CDH1* (E-cadherin), *TP63*, *K14* and *LAMA5* in control, PG/VG and PG/VG+FN groups at different stages of VF epithelial recovery. Error bars with individual data points represent ±s.e.m. of the mean obtained from three biological and technical replicates (*n*=3). (C) Quantitative assessment of cell proliferation. We calculated the percentage of Ki67^+^ cells in VF epithelia and VF fibroblasts, in the control group and experimental groups exposed to PG/VG and PG/VG+FN at different stages of VF epithelial recovery. Error bars with individual data points (average values for each condition) represent ±s.e.m. of the mean. One-way ANOVA with Tukey’s HSD test were used to confirm statistical significance; *P≤0.05.

We extended VF mucosal cultivation for an additional 7 days at the A/Li in plain flavonoid adenine dinucleotide (FAD) medium, which was changed every other day. Our results show that at day 46 (7 days post ECVE exposure), all VF mucosae in the control and experimental groups became thicker (Fig. 6A-C), particularly the PG/VG and PG/VG+FN groups (Fig. 6B,C), and continued to express ECad but with decreased ECad expression in the apical cell layers in the PG/VG and PG/VG+FN groups (Fig. 6D-F). There was also an increase in the number of basal P63^+^ cells in the PG/VG and PG/VG+FN groups. P63^+^ cells became smaller and more tightly packed, causing local basal cell hyperplasia, as compared to the control group (Fig. 6G-I). Moreover, in experimental groups, but not in the controls, we detected an increased K14 signal in epithelial cells (Fig. 6J-L), which was accompanied by local thickening of the LAMA5-positive basement membrane (Fig. 6M-R). To further confirm the histological findings, we performed quantitative analyses and measured the total VF epithelial cross-sectional area and P63^+^ basal cell density and included the early stages of VF epithelial recovery in the analyses (1 and 3 days post ECVE exposure, day 40 and 42 of cell culture, respectively) (Fig. 7A). We established that the total VF epithelial cross-sectional area increased with prolonged cultivation of cells at the A/Li in all groups, resulting in a significant increase in epithelial size and thickness by day 46 (in controls, *P*=0.04798; in PG/VG, *P*=0.01819; and in PG/VG+FN, *P*=0.009798) with the most obvious changes in the PG/VG group. We also observed that the total VF epithelial area gradually increased for the control group, whereas in experimental groups, such as PG/VG and PG/VG+FN, 1 day post ECVE exposure (day 40), we measured a significant drop in the total VF epithelial area (*P*=0.001159). This is likely caused by continuous shedding of damaged luminal cells filled with lipid and/or solvent particles. In addition to the total VF epithelial area, we also measured basal cell density, calculated as the total number of P63^+^ cells per 10,000 µm^2^ (Fig. 7A), which confirmed significant expansion of the P63^+^ basal cell layer at day 42 (3 days post ECVE exposure) in PG/VG (*P*=0.01023) and PG/VG+FN (*P*=0.04829). We also noticed a decreased P63^+^ cell density in control and experimental groups at days 40 and 42 (1 and 3 days post ECVE exposure) compared to days 39 and 46 (Fig. 7A). Except for the control group (*P*=0.0411), these variations in P63^+^ cell density across all time points were not statistically significant and might be related to adjusting of collagen gel-based constructs from submerged conditions back to the A/Li.

Next, we performed qPCR analysis and evaluated the expression levels of the genes expressing structural proteins, i.e. *CDH1*, *TP63*, *K14* and *LAMA5* (Fig. 7B). As expected, we did not observe significant differences in expression of *CDH1* between control and experimental groups across time points, suggesting that, even if the damaged apical cells slough off the surface, the deeper layers continue to express ECad and compensate for the loss of cells to maintain epithelial integrity. Notably, we found significantly upregulated expression of *TP63* at day 40 (*P*<0.00001) in experimental groups versus the control group (Fig. 7B) and significantly increased *K14* (*P*=0.004961) and *LAMA5* (*P*=0.004146) expression that peaked at day 46 (Fig. 7B).

To further investigate whether the increase in *TP63* expression was accompanied by enhanced cell proliferation, we performed a quantitative evaluation of epithelial proliferation (Fig. 7C) and double stained for P63 and Ki67 (Fig. S3A-X). Our results show that the Ki67 signal was primarily detected in the P63^+^ basal cells, as expected (Fig. S3A-X). We calculated the percentage of Ki67^+^ VF epithelial cells (VFEs) and Ki67^+^ VF fibroblasts (VFFs) in the collagen matrix, measuring a significant increase in the proliferation of VFEs in the PG/VG group at day 42 as compared to the control group (*P*=0.01558) (Fig. 7C), followed by a significant decrease at day 46 (*P*=0.0157), which was not observed for the PG/VG+FN group (Fig. 7C). In VF fibroblasts, the number of Ki67^+^ VFFs also peaked at day 42 (3 days post ECVE exposure) in the control and experimental groups; however, due to variations in the number of Ki67^+^ fibroblasts, we observed significant increases in the percentages of Ki67^+^ cells by day 42 in the control group only (*P*=0.01167) (Fig. 7C). These data suggest that lipid-mediated epithelial injury induced reparative processes in the VF mucosae, such as expansion of P63^+^ basal cells during the acute phase of epithelial regeneration (days 40 and 42), followed by active K14 and LAMA5 deposition during the remodeling phase (day 46).

### Reactive epithelial changes are likely regulated by WNT/β-catenin signaling

**Fig. 8.**
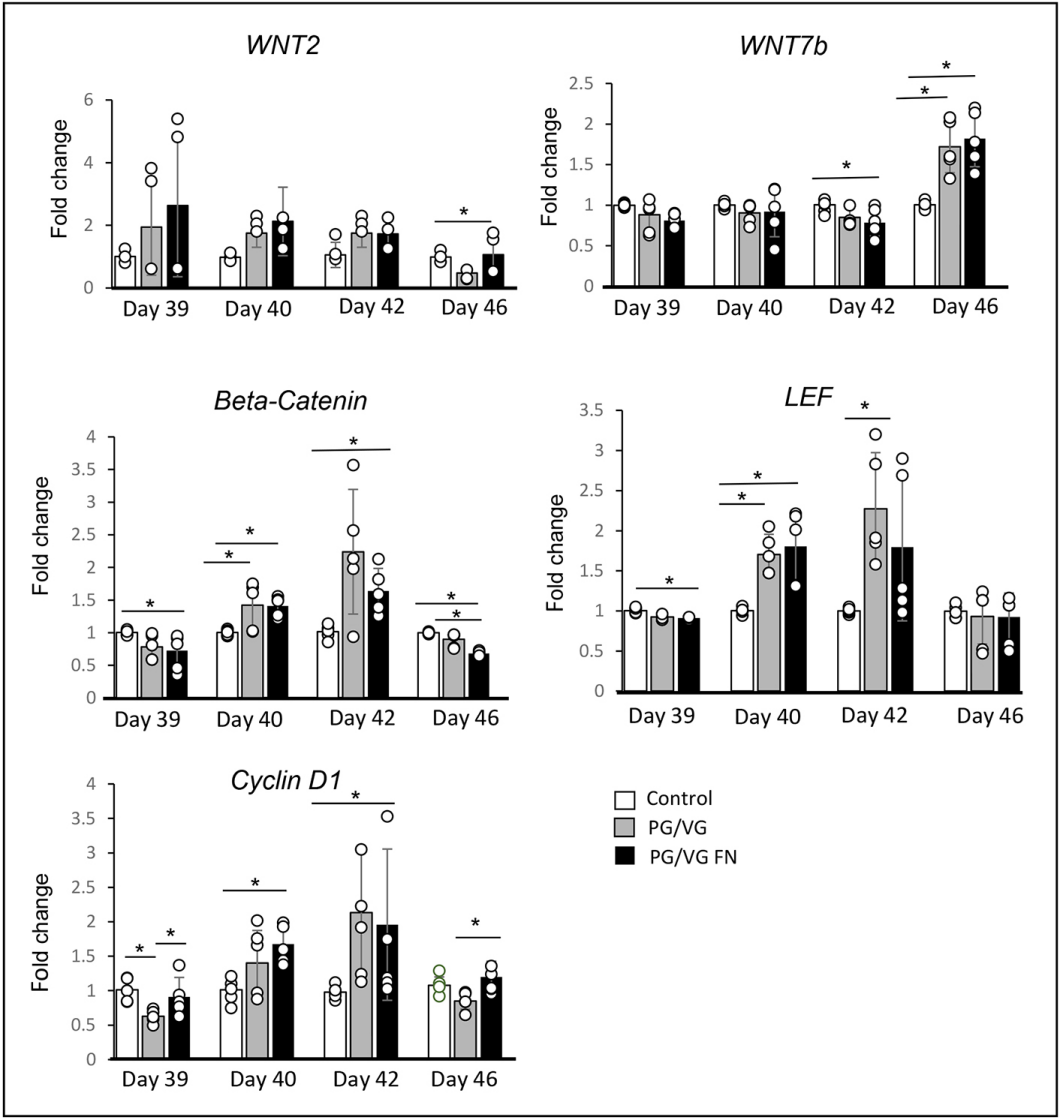
**Transcript levels of members of the WNT/β-catenin signaling pathway after 0.5% ECVE exposure during VF epithelial regeneration.** Transcript levels of *WNT2*, *WNT7B*, *CTNNB1* (β-catenin), *LEF1* and *CCND1* (cyclin D1) in control, PG/VG and PG/VG+FN experimental groups at different stages of VF epithelial recovery. Error bars with individual data points represent ±s.e.m. of the mean obtained from three biological replicates and technical replicates (*n*=3). One-way ANOVA with Tukey’s HSD test were used to confirm statistical significance in gene expression; *P≤0.05.

Lastly, we investigated possible genes and signaling pathways that might control the reparative processes in the VF mucosae in response to ECVE treatment. We performed qPCR, focusing on members of the WNT/β-catenin signaling pathway (Fig. 8). This signaling pathway plays a key role in VF development during embryogenesis in the establishment of VF basal progenitors and VF proliferation (Lungova et al., 2018) and becomes activated postnatally during wound healing in most tissues or organs (Hogan et al., 2014; Morrisey and Hogan, 2010). We found elevated levels of *WNT2* at the early stages of epithelial recovery (days 39 and 40); however, the differences in *WNT2* expression between the experimental and control groups were not statistically significant (Fig. 8). Next, we found a significant 1.4-fold increase in the expression of the β-catenin gene *CTNNB1* in experimental groups versus the control group at day 40 (*P*=0.02212) and a 2.24-fold increase in the PG/VG group and a 1.64-fold increase in the PG/VG+FN group versus the control group at day 42 (*P*=0.02121) (Fig. 8). The significant upregulation of *CTNNB1* was accompanied with a significant increase in lymphoid enhancer-binding factor 1 (*LEF1* or *LEF*) expression at day 40 (1.7-fold change in the PG/VG group and 1.8-fold change in the PG/VG+FN group versus the control group; *P*=0.002237) and at day 42 (2.27-fold change in the PG/VG group and 1.8-fold change in the PG/VG+FN group compared to the control group; *P*=0.032219) (Fig. 8). *CTNNB1*/*LEF1* expression peaked at day 42, which correlates with the peak expression of the cyclin D1 gene *CCND1* (Fig. 8). Cyclin D1 is an important regulator of cell cycle progression and is a direct target of β-catenin (Lungova et al., 2018). At day 40, we found a significant 1.4-fold increase in *CCND1* expression in the PG/VG group and a 1.68-fold increase in the PG/VG+FN group compared to the control group (*P*=0.009682), followed by a significant 2.13-fold increase in the PG/VG group and a 1.96-fold increase in the PG/VG+FN group versus the control group at day 42 (*P*=0.03949) (Fig. 8). At day 46, *CCND1* expression significantly decreased in the PG/VG group versus the control group (*P*=0.001172), which correlates with a significant reduction in the expression of *CTNNB1* and *LEF1* in the experimental groups versus the control group (*P*<0.0001). Notably, we found a significant upregulation of *WNT7B* at day 46 in the experimental groups versus the control group (1.7-fold increase in the PG/VG group and 1.8-fold increase in the PG/VG+FN group; *P*=0.000932) (Fig. 8), which correlates with upregulation of *K14* and *LAMA5.* These findings suggest that WNT7B and the β-catenin/LEF complex can be involved in the regulation of basal cell expansion and epithelial remodeling.

## DISCUSSION

Our recently developed hiPSC model of human VF mucosae holds great promise for studying the toxicity and mechanisms of vaping-related cellular injuries in human VF stratified epithelia. Here, we show that exposure of cells to 0.5% and 5% ECVE for 1 week was sufficient to induce cellular damage in VF apical epithelial cells, which disrupted VF mucosal homeostasis and innate barrier function and triggered epithelial remodeling during VF epithelial recovery. Our results correlate with clinical observations (Bozzella et al., 2020) and previously published animal studies that evaluated the effect of e-cigarette vapor exposure on laryngeal mucosae (Salturk et al., 2015). Rats exposed to e-cigarette vapors for 1 h/day for a period of 4 weeks developed epithelial hyperplasia and metaplasia, accompanied with moderate mucosal inflammation and accumulation of cell debris in the laryngeal lumen as a result of epithelial injury.

In this study, transcriptomic and structural analyses of human VF mucosal cells showed that exposure of cells to ECVE leads to chemical epithelial injury, likely caused by a defective lipid metabolism and the inefficient clearance of lipid/solvent particles that gather in the cytosol and intercellular spaces, as confirmed by Oil Red O staining, TEM and RT^2^ PCR profiling. Lipid particles can be derived from the nicotine and flavorings added to e-liquids to intensify the taste and/or enhance the vaping experience (Chand et al., 2020). We found significant upregulation of *ACADM* in VF mucosae exposed to commercially available e-liquids with nicotine or with nicotine and flavor, supporting the fact that lipid aggregates found in the cell cytoplasm likely contain medium-chain fatty acids/triglycerides that pass through the cell membrane by passive diffusion or via carriers linked to FABP proteins (Schwenk et al., 2010). Medium-chain triglycerides represent a major risk factor associated with vaping along with vitamin E acetate (Blount et al., 2020). Wide testing of commercial e-cigarette products is necessary to identify their potential risks to the individual's health.

We investigated the effect of the lipid-mediated VF epithelial injury on VF mucosal inflammation. We found that ECVE exposure caused moderate VF mucosal inflammation, with significantly upregulated expression of *BMP4* and *BMP7* in the PG/VG group and with significant reduction of *BMP4* and *BMP7* in VF mucosae exposed to PG/VG+N and PG/VG+FN, suggesting that nicotine might suppress BMP production in VF mucosal cells. Previous studies have shown that *BMP4* and *BMP7* are involved in reparative processes, as they are capable of regulating fibroblast and epithelial cell functions. Increased levels of *BMP4* and *BMP7* have been shown to inhibit the differentiation of human lung fibroblasts into myofibroblasts during asthma remodeling and block the production of extracellular matrix proteins by these cells (Pegorier et al., 2010). In epithelial cells, *BMP4* and *BMP7* act on epithelial remodeling in wound-induced epidermal repair, as they regulate keratinocyte proliferation, differentiation, cytoskeletal organization and apoptosis (Lewis et al., 2014). Moreover, the expression levels of BMPs can be influenced by nicotine. In human osteoprogenitor cell cultures obtained from smokers, the transcriptional levels of BMPs, including *BMP4* and *BMP7*, were significantly lower than those in cells derived from non-smokers, significantly delaying fracture healing and bone regeneration (Chassanidis et al., 2012).

Additionally, our results show that nicotine likely stimulates expression of *CCL11* and *IL6* in the PG/VG and PG/VG+FN groups, causing moderate mucosal inflammation*.* Previous studies have demonstrated that the smoking status is associated with elevated levels of *CCL11* in young adults (Greisman et al., 2020) and that cigarette smoke can increase *CCL11* expression and the number of eosinophils in nasal mucosae of young patients with perennial allergic rhinitis (Teixeira et al., 2018; Montaño-Velázquez et al., 2017). The other cytokines that were evaluated were either equally expressed or suppressed upon exposure to ECVE, which corresponds with what has been shown in the literature (Madison et al., 2019; Martin et al., 2016; Higham et al., 2018). However, one of the limitations of this study is the fact that we evaluated cytokine/chemokine expression in VF mucosal cells without the presence of immune cells and macrophages, which could influence their transcript levels. Further validation of our 3D *in vitro* system and the cultivation of VF mucosal cells with a population of immune cells will be extremely useful to provide a comprehensive analysis of the effect of ECVEs on VF mucosal inflammation.

Subsequently, we measured VF mucosal cell responses to ECVE treatment during VF regeneration. We exposed cells to 0.5% ECVE for 1 week to mimic mild but prolonged exposure of VF mucosal cells to vaporized e-liquids. A similar strategy has been used previously in modeling tobacco-related VF diseases (Lungova et al., 2019). The VF epithelium is a stratified squamous epithelium that is composed of a basal cellular compartment, which firmly anchors the epithelium to the basement membrane and provides a reserve of cells necessary for self-renewal (Leydon et al., 2011; Lungova et al., 2019), and suprabasal cells that gradually differentiate as they reach the lumen. Suprabasal and terminally differentiated cells in the apical cell layers face the external environment and perform specific functions, most notably barrier formation, water and ion transport (Levendoski et al., 2014), mucus secretion (Samuels et al., 2008), sensory transduction and immunological surveillance (Leonardi et al., 2015; Günther and Seyfert, 2018). Induced chemical epithelial injury, which removes apical cell layers, compromises the function of the entire epithelial protective barrier. Detached cells wrapped in mucus likely accumulate in the airway lumen, which might contribute to increased throat clearing and coughing in e-cigarette users (Gotts et al., 2019; Bozzella et al., 2020), whereas cells located in the basal cellular compartment initiate remodeling. We detected increased P63^+^ basal cell density at day 42, consistent with the acute phase of mucosal healing, which was confirmed by transcriptome analysis of *TP63* and *CCND1*. Expression levels of both genes culminated at days 40 and 42 (1 and 3 days post ECVE exposure, respectively). We further identified the upregulation of *K14* and *LAMA5*, which peaked at day 46 (7 days post ECVE exposure), exhibited by local thickening of the LAMA5^+^ basement membrane, and increased epithelial keratinization during the remodeling phase.

The VF epithelial responses measured after exposure to ECVE resemble the behavior of VF epithelial cells in reaction to repeated irritations and/or in response to iatrogenic VF injury and appear to be adaptive mechanisms that replace susceptible epithelia with more resistant ones (Ling et al., 2010). In injured porcine and rabbit VFs, epithelial cells undergo proliferation peaking 3 days post injury, which is followed by robust *K14* production and neo-matrix deposition, which culminates between 5 and 14 days post injury (Leydon et al., 2014; Branski et al., 2005). Similarly, murine VF epithelia exposed to high and low doses of cigarette smoke showed enhanced cell proliferation, as confirmed by increased numbers of Ki67^+^ and P63^+^ basal cells, which led to epithelial thickening consistent with hyperplasia (Erickson-DiRenzo et al., 2021). In *in vitro* studies, human VF mucosae exposed to cigarette smoke extract for 1 week enhanced the expression of K14, which accumulated in the luminal cell layers along with K13 (Lungova et al., 2019). Nevertheless, VF mucosal adaptive mechanisms might have adverse consequences on VF health. Repeated episodes of vaping can ultimately cause hyperkeratinization and excessive accumulation of undifferentiated P63^+^ cells in the basal cellular compartment, which might impair epithelial transport efficiency and increase VF mass, thus altering VF vibration and voicing (Erickson-DiRenzo et al., 2021). Histopathological features, such as epithelial hyperplasia, basement membrane thickening and hyperkeratosis, are also found in benign VF lesions (Mostafa et al., 2017), which can develop in response to phonotrauma, and in chemical insults, such as smoking, alcohol abuse or acid reflux (Lechien et al., 2019; Chung et al., 2009). Moreover, persisted epithelial hyperplasia and dysplasia often precede neoplastic VF lesions (Ferlito et al., 2012). Future clinical studies are necessary to confirm whether e-cigarette users are more susceptible to VF diseases compared to conventional cigarette smokers and non-smokers.

Lastly, we investigated the expression of the genes and signaling pathways that might regulate VF mucosal responses to ECVE, focusing on the WNT/β-catenin signaling pathway. We detected elevated transcript levels of *CTNNB1* and *LEF1* and 3 days post ECVE exposure, which correlated with increased expression of *TP63* and *CCND1*, suggesting that the β-catenin/LEF complex might be involved in the regulation of basal cell expansion. Conversely, *WNT7B* expression was activated later, during cytokeratin and extracellular matrix production, suggesting that WNT7B can participate in the regulation of K14 and LAMA5 deposition. These findings are in line with previous investigations. In the airway epithelium, WNT7B induces *FGF10* expression in the underlying mesenchyme, which in turn stimulates cytokeratin production and regeneration of epithelial cells, in response to naphthalene-mediated Clara cell injury (Volckaert et al., 2017). In asthmatic rats, increased transcript levels of *CTNNB1* and *WNT7B* were associated with increased bronchial wall thickness and airway mucosal remodeling (Zhang et al., 2017). Future investigation is warranted to understand the distinct roles of the members of the WNT signaling pathway in laryngeal protection and possible disease development related to vaping and ECVE exposure. Moreover, introduction of short term-ECVE exposures (ranging from 1 to 4 h per day) for an extended period of several weeks will be beneficial to better simulate chronic intermittent episodes of vaping in e-cigarette users.

In summary, this study provides the first comprehensive characterization of human VF epithelial responses to vaping and offers a molecular and cellular foundation for further functional experiments to uncover the genes and signaling pathways that control subsequent VF epithelial remodeling.

## MATERIALS AND METHODS

### Ethical statement

All stem cell work in this investigation was approved by the Stem Cell Research Oversight Committee at the University of Wisconsin-Madison (SC-2015-0008).

### Human iPSC culture and differentiation

For differentiation of hiPSC-derived VF epithelium, we used the commercially available IMR-90-4 cell line transfected with GFP (clone B10), which was validated and screened in our previous study (Lungova et al., 2019). To induce the differentiation of hiPSCs into VF epithelia, we followed our recently published protocol (Lungova et al., 2019). Briefly, IMR-90-4 cells transfected with GFP were maintained in mTeSR1 medium (WiCell, Madison, WI) on plates coated with Matrigel (WiCell, Madison, WI) and were passaged with Versene (STEMCELL Technologies, Vancouver, CA) in a ratio of 1:6. When cells reached 80% confluency, definitive endoderm induction was performed (day 1) using RPMI medium with GlutaMAX (Gibco, Life Technologies) supplemented with 100 ng/ml activin A (Peprotech, Rocky Hill, NJ), 25 ng/ml WNT3a (R&D Systems, Minneapolis, MN) and 10 µM Y-27632 (R&D Systems) for 1 day and RPMI media with GlutaMAX supplemented with 100 ng/ml activin A and 0.2% fetal bovine serum (FBS; Gibco, Life Technologies) for an additional 2 days. At day 4, anterior foregut endoderm (AFE) differentiation was performed. We used Dulbecco's modified Eagle medium (DMEM)/F12 with GlutaMAX (Gibco, Life Technologies) supplemented with N2 and B27 supplements (Gibco, Life Technologies), 0.05 mg/ml ascorbic acid (Millipore, Sigma-Aldrich), 0.4 mM monothioglycerol (Millipore, Sigma-Aldrich) and 1% penicillin-streptomycin (Invitrogen, Carlsbad, CA) (referred to as DMEM basal medium), supplemented with 200 ng/ml noggin (R&D Systems) and 10 µM SB431542 (Tocris, Minneapolis, MN, USA) for 4 days. Medium was changed daily. At Day 8, AFE derived cells were differentiated into VF basal progenitors (VBPs) for 4 days. We used DMEM basal medium supplemented with FGF2 (250 ng/ml), FGF7 (100 ng/ml) and FGF10 (100 ng/ml), all purchased from R&D Systems. The medium was changed every other day. At day 10, VBPs were mildly detached with 0.05% TE (trypsin in EDTA; Gibco, Life Technologies) and transferred to the top of the collagen-fibroblasts constructs to create organotypic VF mucosae.

### Organotypic VF mucosa cultures

One day before VBP re-seeding (day 9), collagen constructs were prepared (Leydon et al., 2013; Lungova et al., 2019) by combining high-concentration rat-tail collagen (4 mg/ml, 80% final volume; BD Biosciences) and 10×DMEM (10% final volume; Millipore, Sigma-Aldrich) on ice and adjusting the pH to 7.2 with 1 N NaOH. VF primary fibroblast 21T cells, passage P5-P6, were resuspended in ice-cold FBS (10% final volume; 500,000 cells/ml final volume) and added to a collagen mixture. A mixture of collagen gel and VF fibroblasts was plated on Transwells (Corning, Millipore, Sigma-Aldrich), 2 ml per a 6-well Transwell, and solidified for 1 h in a tissue incubator at 5% CO_2_ and 37°C. After 1 h, collagen was gently detached with a Pasteur pipette and the constructs were flooded with DMEM basal medium, returned into an incubator and left for 24 h to allow for gel contraction. The next day, VBPs (day 10) were mildly trypsinized and plated on collagen constructs at high density in 100 µl DMEM basal medium supplemented with FGF2 (250 ng/ml), FGF10 (100 ng/ml) and FGF7 (100 ng/ml). Cells were allowed to attach for 2 h and were then flooded with DMEM basal medium with high levels of FGFs, as mentioned above, and cultivated for 2 days to complete VBP differentiation (day 12). On day 12, DMEM basal medium was changed for conditional FAD medium (prepared as described below) supplemented with high levels of FGFs and VBPs were further differentiated as submerged cultures for 2 days. At day 14, the conditional culture medium was aspired from the upper inserts and cells were cultivated at the A/Li. The A/Li culture was performed in conditional FAD medium with FGFs for the first 4 days and plain FAD medium for an additional 2 weeks. FAD medium was freshly prepared every week. It consisted of DMEM and F12 in a ratio of 1:3 (Gibco, Life Technologies), supplemented with 2.5 ml FBS, 0.4 µg/ml hydrocortisone (Millipore, Sigma-Aldrich) 8.4 ng /ml cholera toxin (Millipore, Sigma-Aldrich), 5 µg/ml insulin (Millipore, Sigma-Aldrich), 24 µg/ml adenine (Millipore, Sigma-Aldrich), 10 ng/ml epidermal growth factor (R&D Systems) and 1% penicillin-streptomycin (Invitrogen). In submerged cultures, 1 ml of FAD was applied on Transwells with collagen constructs and 2 ml was applied in the basolateral chamber. FAD was changed every other day. To create the A/Li, FAD medium was placed in the basolateral chamber only and changed three times a week. Conditional FAD medium was formed by cultivation of FAD with human primary VFF 21T cells for 24 h at 37°C with 5% CO_2_. After 24 h, the medium was collected, sterile filtered and stored at −20°C. The ratio of 30:70 (30% for conditional and 70% for fresh FAD medium) was used in the experiment.

### Preparation of the electronic cigarette vapor extract (ECVE)

ECVE (100%) was generated as recently described (Lungova et al., 2019). Briefly, e-cigarette vapors (sold by Infinite Vapor, infinitevapor.com) were bubbled through 30 ml of the DMEM/F12 medium in a disposable 50 ml tube with the use of an experimenter-operated syringe. Human vaping was modeled with two short puffs (2 s) with long delays between puffs (30 s). Two short puffs were repeated 15 times (30 puffs total), which was equivalent to three conventional cigarettes as used in our previous study (Lungova et al., 2019) for each experimental condition. Aerosolized vapors that were drawn through the end of the e-cigarette during vaping were bubbled through the DMEM medium. The obtained medium was considered 100% EVCE. To ensure standardization between experiments, ECVE was sterile-filtered through a 0.2 mm filter, aliquoted and stored at −80°C. Before usage, ECVE was quickly thawed and diluted with the FAD medium to the indicated concentration and used the same day. We prepared three different 100% ECVE extracts: PG/VG only (vehicle control) (Hell Vapors; batch number: G0391); PG/VG with 1.8% nicotine (Hells Vapors, batch number: C1291) and PG/VG with 1.2% nicotine and Unicorn Poop flavor (Drip Star, batch number: E0891).

### Exposure of VF mucosae to 0.5% and 5% EVCE

To create the lipid-mediated VF injury, inserts (upper chamber) containing engineered VF mucosae at day 32 of differentiation were flooded with FAD medium supplemented with 0.5% or 5% ECVE for 1 week. The lower chamber was flooded with plain FAD medium. For 0.5% ECVE, we included two experimental groups: PG/VG (vehicle control) and PG/VG+FN. For 5% ECVE, we included three experimental groups: PG/VG, PG/VG+N and PG/VG+FN. Engineered VF mucosae flooded with plain FAD medium in the upper and lower chambers were used as negative controls. The medium was changed every day in both chambers. After 1 week of exposure to the 0.5% or 5% ECVE, engineered VF mucosae were harvested and characterized with immunohistochemistry (IHC), RT^2^ PCR profiling and qPCR to investigate the expression levels of clinically relevant genes. We also performed Oil Red O staining on frozen VF mucosal sections to detect lipid particles and TEM to evaluate the ultrastructure of VF apical epithelial cell layers. In VF mucosae exposed to 0.5% ECVE, we extended the *in vitro* cultivation for an additional week to assess the effect of ECVE exposure on VF epithelial regeneration. At day 39, after 1 week of exposure to the 0.5% ECVE, we aspired old FAD medium and cultivated engineered VF mucosae at A/Li for an additional 1, 3 and 7 days. We applied plain FAD medium into the lower chambers only, in both the control and experimental groups, and replaced the medium every other day. At the selected time points, VF mucosae were harvested and characterized by IHC and qPCR.

### Cryosectioning and Oil Red O staining

At day 39, the medium was aspired from the control and 5% ECVE-exposed VF mucosae; 3D constructs were briefly washed in PBS immediately and embedded in separate molds with optimal cutting temperature mounting medium (Sakura, Hayward, CA) and placed on dry ice to freeze tissues. Blocks were stored in the freezer at −80°C. Before cryosectioning, blocks were removed from the freezer, allowed to warm up to −21°C in a cryostat (Leica, CM3050S) and cut to 5 µm-thick sections (chamber and objective temperatures of −21°C). Sections were collected on pre-coated slides and immediately used for Oil Red O staining using the Oil Red O Stain Kit (Lipid Stain) (Abcam, Cambridge, UK) following the manufacturer's protocol. Samples were counterstained with Hematoxylin and mounted with Cytoseal TM XYL (Thermo Fisher Scientific). Images were taken with a Nikon Eclipse E600 with an Olympus DP71 camera and were adjusted for brightness using the installed DP 71 software (Olympus Corporation).

### IHC of collagen gel constructs

At the selected time points (days 39, 40, 42 and 46), collagen gel constructs were harvested. For IHC staining, we first washed the constructs in PBS, fixed them in fresh 4% paraformaldehyde for 15 min at room temperature and embedded them in HistoGel (Thermo Fisher Scientific). The constructs were then dehydrated in ethanol, treated with xylene, embedded in paraffin and cut to 5 µm-thick serial sections. Sections were then deparaffinized, rehydrated and stained using a standard IHC protocol (Lungova et al., 2019). Antigen retrieval was performed by heating sections in sodium citrate, pH 6, in an 80°C water bath for 2 h. All primary and secondary antibodies in this study have been routinely used before and validated elsewhere (Lungova et al., 2019). The following primary antibodies were used: rabbit anti-laminin α-5 (1:100, Abcam, ab11575), anti-rabbit cytokeratin 14 (1:250, ProteinTech, 10143-1-AP), anti-rabbit cytokeratin 13 (1:100, Abcam, ab97327), anti-rabbit E-cadherin (1:100, Cell Signaling Technology, 3195S), anti-mouse P63 (1:100, Biocare Medical, CM143A), anti-mouse MUC1 (1:200, Abcam, ab70475), anti-mouse MUC4 (1:200, Abcam, ab60720) and anti-rabbit Ki67 (1:200, Abcam, ab6667). The following secondary antibodies were used: Alexa Fluor 488 goat anti-rabbit (1:500, Invitrogen, A11070) and Cy3-conjugated goat anti-mouse (1:200, Jackson ImmunoResearch, 115-165-003). The samples were incubated in primary antibodies for 24 h at 4°C and in secondary antibodies for 1 h 30 min at room temperature. Slides were mounted using Vectashield with DAPI (Vector Laboratories; Peterborough, UK). Images were acquired with a Nikon Eclipse Ti2 and were adjusted for brightness using the installed NIS-Elements software (Nikon). Qualitative experiments were not blinded and were repeated twice in the laboratory by two investigators. We used three biological and two technical replicates in the procedure (*n*=3) for control and experimental groups and for each time point.

### Quantitative assessment of total VF epithelial area and P63^+^ basal cell density

P63^+^- and DAPI-labeled cross-sections collected at 0, 1, 3 and 7 days post ECVE exposure (days 39, 40, 42 and 46, respectively) were analyzed using ImageJ software. The VF cross-sectional area was outlined and measured using the ImageJ area measurement tool. Simultaneously, we manually counted P63^+^ cells and calculated P63 cell density, presented as the number of P63^+^ basal cells per 10,000 µm^2^. For each control and experimental condition, time point and biological replicate, ten images were analyzed (*n*=3; 360 images total). The images were taken at 40× magnification from the left edge of the construct, through the middle portion and towards the right edge of the construct to characterize changes in the VF epithelium along the entire length of the construct. One-way ANOVA with Tukey’s honestly significant difference (HSD) test were used to confirm statistical significance in total VF epithelial cross-sectional areas and P63^+^ basal cell density, *P≤0.05.

### Quantitative assessment of cell proliferation

Ki67^+^- and DAPI-labeled VFEs and VFFs collected at 0, 1, 3 and 7 days post ECVE exposure (days 39, 40, 42 and 46, respectively) were manually counted using the ImageJ cell counting tool. For each control and experimental condition, time point and biological replicate, ten images were analyzed (*n*=3; 360 images total). As stated above, the images were taken at 40× magnification from the left edge of the construct, through the middle portion and towards the right edge of the construct to characterize changes in cell proliferation along the entire length of the construct. The percentages of Ki67^+^ nuclei in VFEs and VFFs in control and experimental groups at different stages of VF regeneration were compared using one-way ANOVA with Tukey's HSD test to confirm statistical significance, *P≤0.05.

All quantitative analyses were performed as blinded experiments. VF mucosal histological sections were randomly assigned with numbers ranging from one to 36, then they were imaged (ten images per number using the strategy described above). We performed inter-rater reliability on 20% of dependent variables for VF epithelial cross-sectional area measurement, P63^+^ basal cell density and quantitative assessment of cell proliferation in the percentage of Ki67^+^ VFEs and VFFs. The first eight datasets (20%) were counted independently by two raters and Pearson' correlation coefficient was calculated to determine the level of agreement (VF cross-sectional area, R=0.9919; P63^+^ cell density, R=0.9845; percentage of Ki67^+^ VFEs, R=0.9071; percentage of Ki67^+^ VFFs, R=0.7536).

### TEM

For TEM, gels were fixed in 2.5% glutaraldehyde and 2% paraformaldehyde in a 0.1 M sodium cacodylate buffer (pH 7.4) overnight at 4°C and processed using routine techniques. Briefly, gels were washed in a 0.1 M sodium cacodylate buffer and postfixed in 1% osmium tetroxide in the same buffer for 2 h at room temperature. Tissues were dehydrated in graded ethanol series, rinsed twice in propylene oxide and embedded in Epon 812 epoxy resin (Polysciences, Warrington, PA) under vacuum. Finally, the samples were flat embedded between glass slides. After resin polymerization, one of the two glass slides was removed and blank resin cylinders were glued to the sections. The gels were thin sectioned for TEM using a Leica EM UC6 Ultramicrotome (90 nm-thick sections) and stained with Reynold's lead citrate and 8% uranyl acetate in 50% ethanol to increase contrast. The sections were viewed with a Philips CM120 electron microscope and images were captured with a MegaView III side-mounted digital camera.

### Isolation of VF mucosal cells for RNA isolation

To isolate populations of VF mucosal cells from the whole constructs, collagen gel was first dissolved using collagenase (Gibco Collagenase, Type I, Powder; 17018029, Gibco). Briefly, old medium was aspired and constructs were washed twice in PBS. Collagenase type I at a working concentration of 100 U/ml was added to the upper (1 ml) and lower chambers (2 ml), with the VF mucosae being fully submerged. Constructs were incubated at 37°C for at least 2-3 h, or until the collagen completely dissolved and cells were loosened. The cell suspension was then transferred into a 15 ml conical tube. Cells were centrifuged for 5 min at 200 ***g***, washed and resuspended in PBS and transferred into a 1.5 ml tube. Cells were again centrifuged for 5 min at 200 ***g***, the supernatant was aspired and cell pellet was stored at −80°C.

### RNA isolation and qPCR

Cells isolated from whole constructs were used for RNA isolation using ReliaPrep RNACell Miniprep System (Promega, Madison, WI) according to the manufacturer's protocol. RNA (1000 ng; or 500 ng for low-yield RNA samples) was reverse transcribed to cDNA using reverse transcription reagents (Go Script, Promega, Madison, WI) as per the manufacturer's protocol. A total volume of 0.4 µl of cDNA (0.8 µl of cDNA for low-yield RNA/cDNA samples) was used per 20 µl real-time qPCR reaction using Power Up SYBR Green Master Mix (Applied Biosystems, Foster City, CA) and run for 40 cycles in triplicates on a 7500 Fast Real Time PCR System machine (Applied Biosystems), according to the manufacturer's instructions. Gene-specific primers are listed in Table S7. Relative gene expression, normalized to β-actin (ΔCT) and control VF mucosae (ΔΔCT), was calculated as fold change using the 2^−ΔΔCT^ method. If undetected, a cycle number of 40 was assigned to allow fold change calculations. Data are presented as the average of the three biological and technical replicates (for 5% ECVE) and three biological and technical replicates (for 0.5% ECVE) ±s.e.m. of the mean. One-way ANOVA with Tukey’s HSD test were used to confirm statistical significance in gene expression, *P≤0.05.

### RT^2^ PCR profiling

Cells isolated from whole constructs were used for RNA isolation using ReliaPrep RNACell Miniprep System (Promega) according to the manufacturer's protocol. RNA (500 ng) was reverse transcribed to cDNA using a reverse transcription RT^2^ First Strand Kit (QIAGEN, Hilden, Germany) according to the manufacturer's protocol. Then, we performed RT^2^ Profiler PCR Arrays. The cDNA was first diluted with nuclease-free water and then added to the RT^2^ SYBR Green ROX qPCR Mastermix (QIAGEN) according to the manufacturer's protocol. We used RT^2^ Profiler PCR Arrays for human cytokines and chemokines (PAHS-150Z, QIAGEN) and fatty acid metabolism (PAHS-007Z, QIAGEN). A RT^2^ Profiler PCR Array Format E 384 (4×96) was used; it contained four replicate primer assays for each of the 84 pathway-focused genes and five replicate primer assays for each of the housekeeping genes used as a reference, i.e. β-actin (*ACTB*), β-2-microglobulin (*B2M*), glyceraldehyde-3-phosphate dehydrogenase (*GAPDH*), hypoxanthine phosphoribosyltransferase 1 (*HPRT1*) and ribosomal protein, large, P0 (*RPLP0*). The control and each test group were run in triplicate (*n*=3, 12 samples total). We used a real-time cycler Quant Studio 5 (Applied Biosystems). The cycling conditions were as follows: stage 1, 95°C for 10 mins; stage 2, 95°C for 15 s followed by 60°C for 1 min with 40 repeats (40 cycles); and stage 3 (dissociation), 95°C for 15 s followed by 60°C for 1 min, then 95°C for 15 s followed by 60°C for 15 s. CT values were exported to an Excel file (Tables S2 and S5). These tables were then uploaded to the data analysis web portal at http://www.qiagen.com/geneglobe. Samples were assigned to control and test groups: PG/VG (group 1), PG/VG+N (group 2) and PG/VG+FN (group 3). The CT cut-off was set to 35. CT values were normalized based on a manual selection of reference (housekeeping) genes. The data analysis web portal calculated fold change/regulation using the ΔΔCT method, in which ΔCT was calculated as the difference in expression between the gene of interest and an average of the expression of reference genes, followed by ΔΔCT calculations [ΔCT (test group)/ΔCT (control group)] (Tables S3 and S6). The error bar calculations were not included in the final reports by QIAGEN, therefore, the results are presented without the error bars. The fold-change threshold was set to 2. The *P*-values were calculated based on a two-tailed unpaired Student's *t*-test of the replicate 2^−ΔΔCT^ values for each gene in the control group and treatment groups and *P*<0.05 was considered as significant. The *P*-value calculation was based on parametric, unpaired, two-sample equal variance and two-tailed distribution.

### Estimating sample size and power analysis

All histology analyses including quantitative assessment of total VF epithelial area, P63^+^ basal cell density, and cell proliferation and qPCR analyses were performed in three biological replicates. We determined sample size built on our previous work using the same experimental system (Lungova et al., 2019). Based on a two-tailed two-sample *t*-test at an α level of 0.05 significance, three biological replicates per group yielded a power of 80% to detect a significant difference in gene expression between controls and experimental groups. For statistical analyses, one-way ANOVA with Tukey’s HSD test for pairwise comparisons were used; *P*≤0.05 confirmed statistical significance.

## Supplementary Material

Supplementary information
