# Supplementary material for: Exposure to e-cigarette vapor extract induces vocal fold epithelial injury and triggers intense mucosal remodeling
Source: Dis Model Mech. 2022 Aug 23;15(8):dmm049476. doi: 10.1242/dmm.049476 (PMC9438930; doi:10.1242/dmm.049476)
Supplement: Supplementary information [file dmm-15-049476-s1.pdf]

Supplementary Figure S1

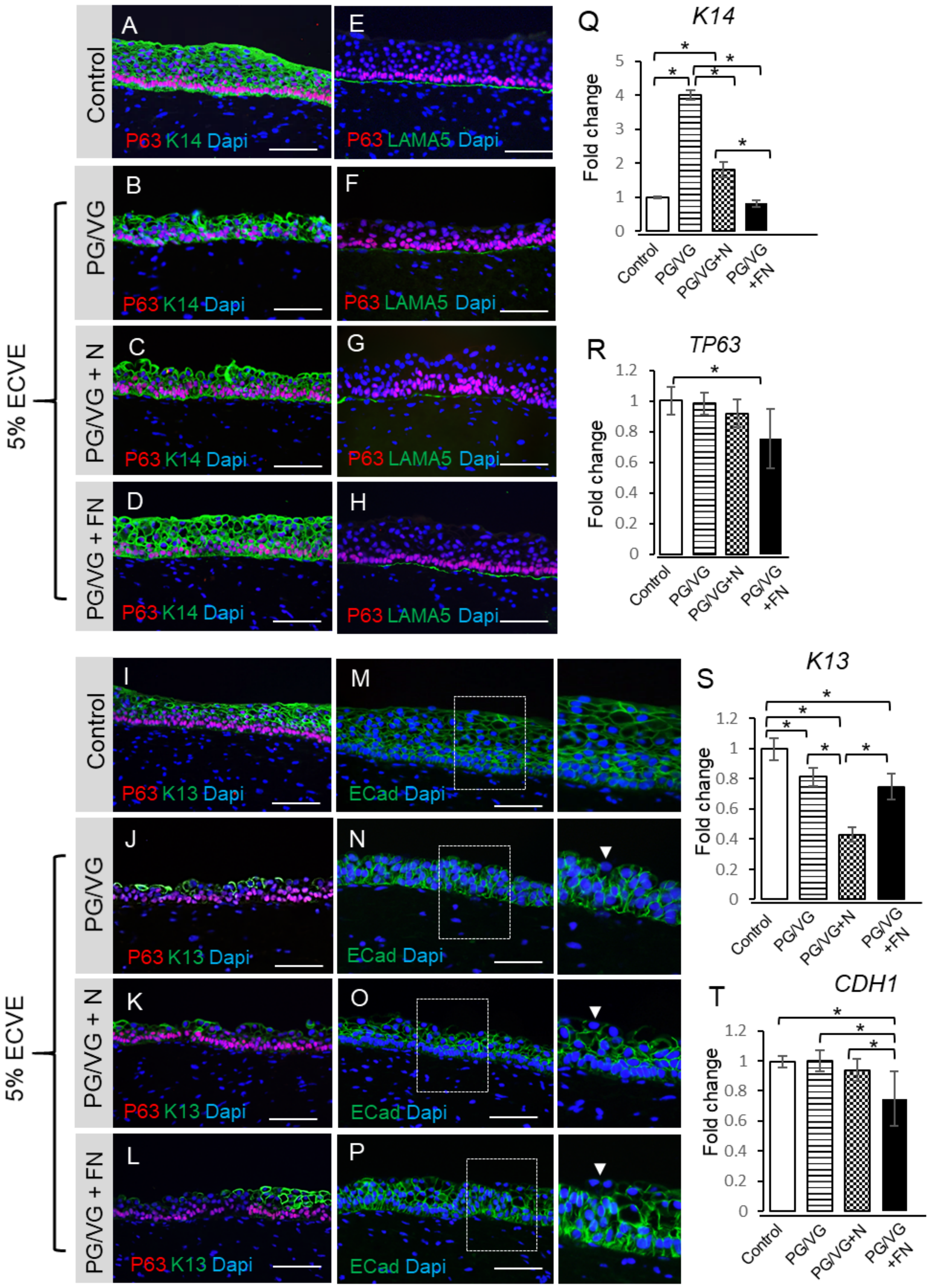

Disease Models & Mechanisms • Supplementary information

Fig. S1. Distribution and expression levels of VF structural epithelial genes. (A-D) Anti-Cytokeratin 14 (green) and anti-P63 staining (red) in control (A) and 5% ECVE exposed VF mucosae (B-D). (E-H) Anti-Laminin alfa 5 (green) co-stained with anti-P63 (red) in control (E) and 5% ECVE treated VF mucosae (F-H). (I-L) Anti-Cytokeratin 13 (in green) co-stained with anti-P63 (in red) in control (I) and 5% ECVE exposed VF mucosae (J-L). (M-P) Anti-E-Cadherin staining (in green) in control (M) and 5% ECVE exposed VF mucosae (N-P). Bracketed regions in the panels of M, N, O and P are magnified in the boxes on the right. White arrows point to apical cells with decreased expression of E-Cadherin. Scale bars = 100μm. (Q-T) Transcript levels of *KRT14* (Q), *TP63* (R), *KRT13* (S) and *CDH1* (T) in control and 5% ECVE exposed VF mucosae. Error bars represent ± standard error of the mean obtained from three biological and three technical replicates. All histological data were performed in three biological replicates (n=3) and repeated twice in the laboratory by two investigators. One-Way ANOVA of variance for independent or correlated samples statistical analysis along with Plus Tukey HSD test were used to confirm statistical significance in gene expression, p-Value ≤ 0.05 (*).

Supplemental Figure S2

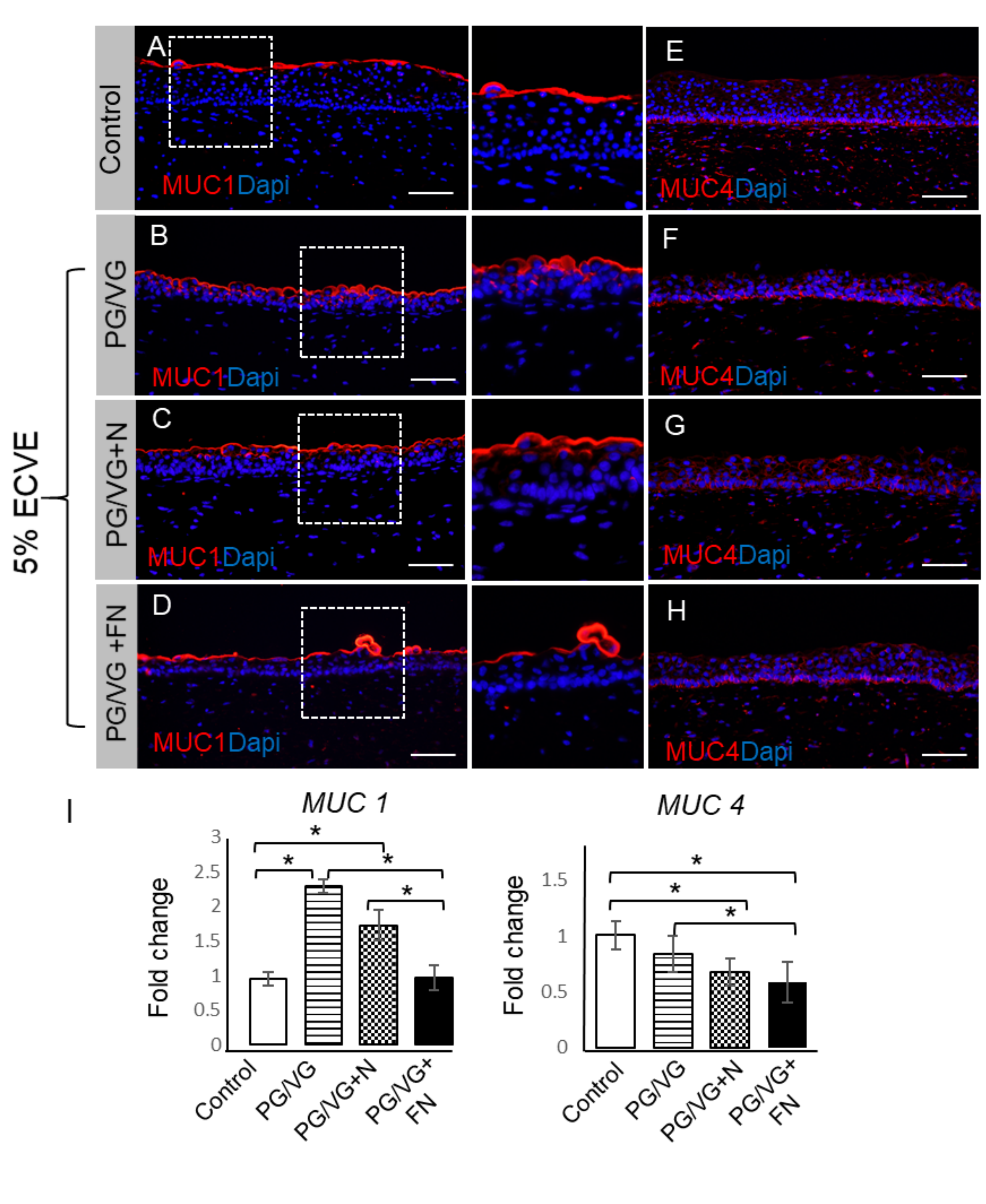

Fig. S2. Distribution and expression levels of functional VF epithelial genes. (A-D) Red immunofluorescent staining of Mucin 1 (MUC1) in control (A) and 5% ECVE exposed VF mucosae (B-D). Bracketed regions in the panels of A-D are magnified on the right respectively. (E-H) Red immunofluorescent staining of Mucin 4 (MUC4) in control (E) and 5% ECVE treated VF mucosae (F-H). Scale bar = 100μm. All histological data were performed in three biological replicates (n=3) and repeated twice in the laboratory by two investigators (I) Transcript levels of *MUC 1* and *MUC 4.* Error bars represent ± standard error of the mean obtained from three biological and three technical replicates. One-Way ANOVA of variance for independent or correlated samples statistical analysis along with Plus Tukey HSD test were used to confirm statistical significance in gene expression, p-Value ≤ 0.05 (*).

Disease Models & Mechanisms • Supplementary information

Supplementary Figure S3

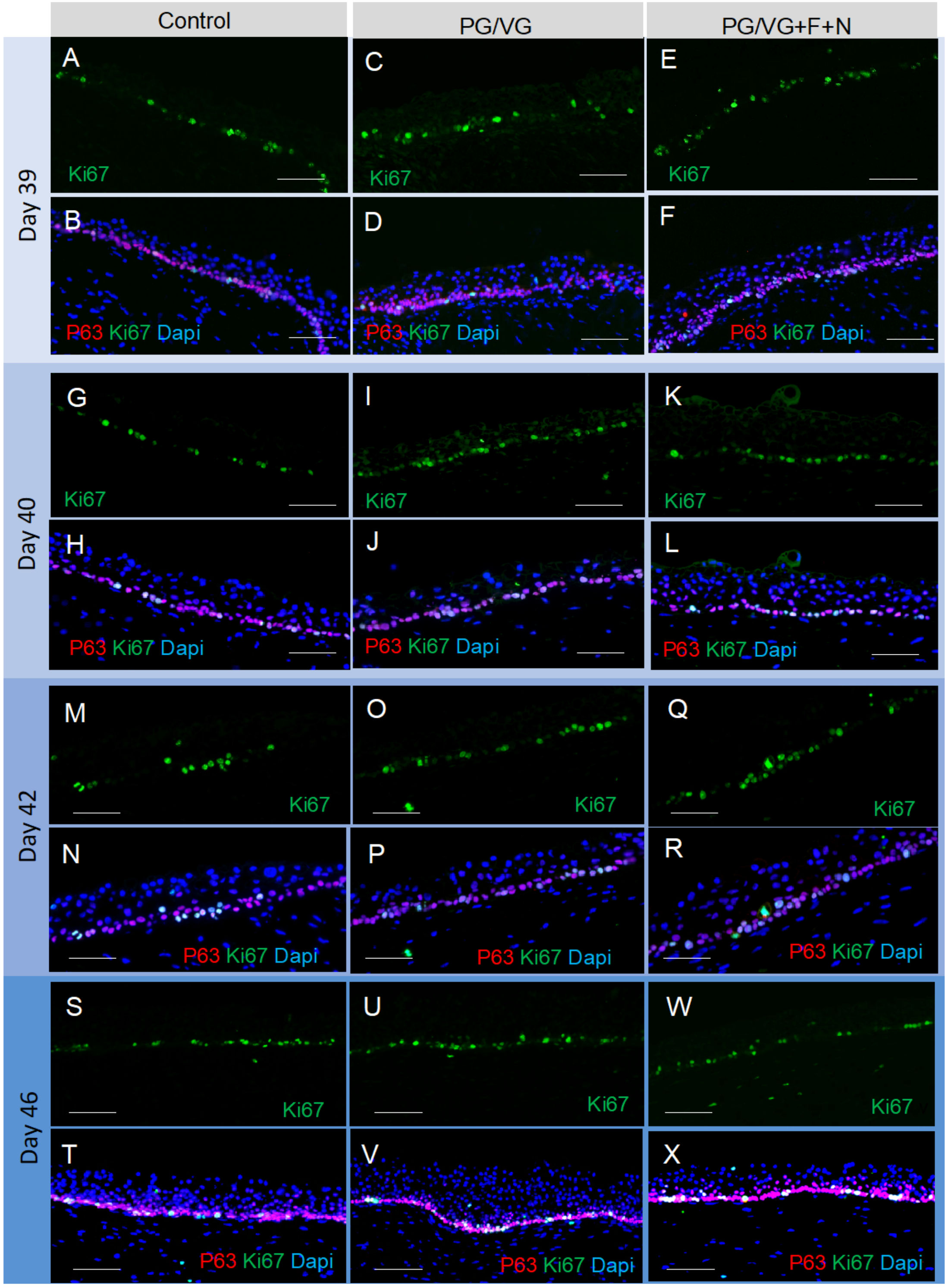

Fig. S3. Assessment of cell proliferation in control and 0.5% ECVE exposed VF mucosae at different stages of VF epithelial recovery (A -X). Anti-Ki67 staining (green) and anti-Ki67 co-stained with P63 (red) in control and 0.5% ECVE exposed VF mucosae at Day 39 (A-F), at Day 40 (G-L), at Day 42 (M-R), and at Day 46 (S-X). Scale bar = 100µm. All histological data were performed in three biological replicates (n=3) and repeated twice in the laboratory by two investigators

Disease Models & Mechanisms • Supplementary information

Table S1. Genes analyzed in the RT^2 Profiler PCR Arrays --Human Cytokine and Chemokines (PAHS-150Z).

Position	RefSeq Number	**Symbol**	Description
A01	NM_004797	**ADIPOQ**	Adiponectin, C1Q and collagen domain containing
A02	NM_001200	**BMP2**	Bone morphogenetic protein 2
A03	NM_130851	**BMP4**	Bone morphogenetic protein 4
A04	NM_001718	**BMP6**	Bone morphogenetic protein 6
A05	NM_001719	**BMP7**	Bone morphogenetic protein 7
A06	NM_001735	**C5**	Complement component 5
A07	NM_002981	**CCL1**	Chemokine (C-C motif) ligand 1
A08	NM_002986	**CCL11**	Chemokine (C-C motif) ligand 11
A09	NM_005408	**CCL13**	Chemokine (C-C motif) ligand 13
A10	NM_002987	**CCL17**	Chemokine (C-C motif) ligand 17
A11	NM_002988	**CCL18**	Chemokine (C-C motif) ligand 18 (pulmonary and activation-regulated)
A12	NM_006274	**CCL19**	Chemokine (C-C motif) ligand 19
B01	NM_002982	**CCL2**	Chemokine (C-C motif) ligand 2
B02	NM_004591	**CCL20**	Chemokine (C-C motif) ligand 20
B03	NM_002989	**CCL21**	Chemokine (C-C motif) ligand 21
B04	NM_002990	**CCL22**	Chemokine (C-C motif) ligand 22
B05	NM_002991	**CCL24**	Chemokine (C-C motif) ligand 24
B06	NM_002983	**CCL3**	Chemokine (C-C motif) ligand 3
B07	NM_002985	**CCL5**	Chemokine (C-C motif) ligand 5
B08	NM_006273	**CCL7**	Chemokine (C-C motif) ligand 7

Disease Models & Mechanisms • Supplementary information

B09	NM_005623	**CCL8**	Chemokine (C-C motif) ligand 8
B10	NM_000074	**CD40LG**	CD40 ligand
B11	NM_000614	**CNTF**	Ciliary neurotrophic factor
B12	NM_000757	**CSF1**	Colony stimulating factor 1 (macrophage)
C01	NM_000758	**CSF2**	Colony stimulating factor 2 (granulocyte-macrophage)
C02	NM_000759	**CSF3**	Colony stimulating factor 3 (granulocyte)
C03	NM_002996	**CX3CL1**	Chemokine (C-X3-C motif) ligand 1
C04	NM_001511	**CXCL1**	Chemokine (C-X-C motif) ligand 1 (melanoma growth stimulating activity, alpha)
C05	NM_001565	**CXCL10**	Chemokine (C-X-C motif) ligand 10
C06	NM_005409	**CXCL11**	Chemokine (C-X-C motif) ligand 11
C07	NM_000609	**CXCL12**	Chemokine (C-X-C motif) ligand 12
C08	NM_006419	**CXCL13**	Chemokine (C-X-C motif) ligand 13
C09	NM_022059	**CXCL16**	Chemokine (C-X-C motif) ligand 16
C10	NM_002089	**CXCL2**	Chemokine (C-X-C motif) ligand 2
C11	NM_002994	**CXCL5**	Chemokine (C-X-C motif) ligand 5
C12	NM_002416	**CXCL9**	Chemokine (C-X-C motif) ligand 9
D01	NM_000639	**FASLG**	Fas ligand (TNF superfamily, member 6)
D02	NM_000175	**GPI**	Glucose-6-phosphate isomerase
D03	NM_000605	**IFNA2**	Interferon, alpha 2
D04	NM_000619	**IFNG**	Interferon, gamma
D05	NM_000572	**IL10**	Interleukin 10
D06	NM_000641	**IL11**	Interleukin 11
D07	NM_000882	**IL12A**	Interleukin 12A (natural killer cell stimulatory factor 1, cytotoxic lymphocyte maturation factor 1, p35)
D08	NM_002187	**IL12B**	Interleukin 12B (natural killer cell stimulatory factor 2, cytotoxic lymphocyte maturation factor 2, p40)

D09	NM_002188	**IL13**	Interleukin 13
D10	NM_000585	**IL15**	Interleukin 15
D11	NM_004513	**IL16**	Interleukin 16
D12	NM_002190	**IL17A**	Interleukin 17A
E01	NM_052872	**IL17F**	Interleukin 17F
E02	NM_001562	**IL18**	Interleukin 18 (interferon-gamma-inducing factor)
E03	NM_000575	**IL1A**	Interleukin 1, alpha
E04	NM_000576	**IL1B**	Interleukin 1, beta
E05	NM_000577	**IL1RN**	Interleukin 1 receptor antagonist
E06	NM_000586	**IL2**	Interleukin 2
E07	NM_021803	**IL21**	Interleukin 21
E08	NM_020525	**IL22**	Interleukin 22
E09	NM_016584	**IL23A**	Interleukin 23, alpha subunit p19
E10	NM_006850	**IL24**	Interleukin 24
E11	NM_145659	**IL27**	Interleukin 27
E12	NM_000588	**IL3**	Interleukin 3 (colony-stimulating factor, multiple)
F01	NM_000589	**IL4**	Interleukin 4
F02	NM_000879	**IL5**	Interleukin 5 (colony-stimulating factor, eosinophil)
F03	NM_000600	**IL6**	Interleukin 6 (interferon, beta 2)
F04	NM_000880	**IL7**	Interleukin 7
F05	NM_000584	**CXCL8**	Interleukin 8
F06	NM_000590	**IL9**	Interleukin 9
F07	NM_002309	**LIF**	Leukemia inhibitory factor (cholinergic differentiation factor)
F08	NM_000595	**LTA**	Lymphotoxin alpha (TNF superfamily, member 1)
F09	NM_002341	**LTB**	Lymphotoxin beta (TNF superfamily, member 3)
F10	NM_002415	**MIF**	Macrophage migration inhibitory factor (glycosylation-inhibiting factor)

Disease Models & Mechanisms • Supplementary information

F11	NM_005259	**MSTN**	Myostatin
F12	NM_018055	**NODAL**	Nodal homolog (mouse)
G01	NM_020530	**OSM**	Oncostatin M
G02	NM_002704	**PPBP**	Pro-platelet basic protein (chemokine (C-X-C motif) ligand 7)
G03	NM_000582	**SPP1**	Secreted phosphoprotein 1
G04	NM_003238	**TGFB2**	Transforming growth factor, beta 2
G05	NM_000460	**THPO**	Thrombopoietin
G06	NM_000594	**TNF**	Tumor necrosis factor
G07	NM_002546	**TNFRSF11B**	Tumor necrosis factor receptor superfamily, member 11b
G08	NM_003810	**TNFSF10**	Tumor necrosis factor (ligand) superfamily, member 10
G09	NM_003701	**TNFSF11**	Tumor necrosis factor (ligand) superfamily, member 11
G10	NM_006573	**TNFSF13B**	Tumor necrosis factor (ligand) superfamily, member 13b
G11	NM_003376	**VEGFA**	Vascular endothelial growth factor A
G12	NM_002995	**XCL1**	Chemokine (C motif) ligand 1
H01	NM_001101	**ACTB**	Actin, beta
H02	NM_004048	**B2M**	Beta-2-microglobulin
H03	NM_002046	**GAPDH**	Glyceraldehyde-3-phosphate dehydrogenase
H04	NM_000194	**HPRT1**	Hypoxanthine phosphoribosyltransferase 1
H05	NM_001002	**RPLP0**	Ribosomal protein, large, P0
H06	SA_00105	**HGDC**	Human Genomic DNA Contamination
H07	SA_00104	**RTC**	Reverse Transcription Control
H08	SA_00104	**RTC**	Reverse Transcription Control
H09	SA_00104	**RTC**	Reverse Transcription Control
H10	SA_00103	**PPC**	Positive PCR Control
H11	SA_00103	**PPC**	Positive PCR Control
H12	SA_00103	**PPC**	Positive PCR Control

Disease Models & Mechanisms • Supplementary information

Table S2. List of CT values of human cytokines and chemokines uploaded on to the data analysis web portal at http://www.qiagen.com/geneglobe

	Control Group	Control Group	Control Group	PG/VG Test Group 1	PG/VG Test Group 1	PG/VG Test Group 1	PG/VG+N Test Group 2	PG/VG+N Test Group 2	PG/VG+N Test Group 2	PG/VG+N+F Test Group 3	PG/VG+N+F Test Group 3	PG/VG+N+F Test Group 3
A01	34.69	35.26	33.19	32.75	35.37	33.31	34.02	34.20	33.79	34.18	34.83	34.92
A02	26.76	30.27	27.15	29.55	29.60	26.59	27.40	28.55	27.19	27.15	29.63	26.95
A03	27.22	28.81	27.23	25.69	26.43	23.47	25.81	28.06	27.14	26.52	28.30	26.62
A04	28.82	29.82	28.60	29.21	28.57	26.89	26.97	28.59	28.68	27.31	28.92	27.74
A05	28.90	28.23	26.85	26.13	26.45	24.38	26.76	28.28	27.24	28.05	30.05	28.34
A06	28.75	40	28.14	30.70	40	27.85	27.24	28.81	29.23	26.98	28.71	27.65
A07	30.55	40	32.39	30.43	30.58	29.29	29.08	30.20	29.47	28.94	29.81	29.82
A08	32.12	33.44	31.04	34.58	31.63	30.66	27.92	29.42	29.51	28.08	30.06	28.46
A09	31.70	31.66	30.35	30.38	30.40	29.59	28.64	30.60	29.81	29.84	29.61	29.07
A10	33.58	33.11	33.38	32.00	34.11	31.91	33.22	34.29	32.55	34.68	33.79	32.24
A11	32.61	30.82	32.93	31.83	30.22	31.36	32.89	29.98	31.27	33.59	30.44	33.80
A12	37.71	35.72	31.67	34.27	32.71	31.91	33.47	31.44	35.16	36.19	31.72	33.57
B01	26.31	27.03	25.41	24.86	24.83	23.13	23.62	25.97	24.01	24.33	26.58	24.55
B02	27.14	27.90	27.27	27.90	27.18	25.89	26.11	27.45	26.32	25.53	26.29	25.60
B03	31.60	33.37	32.96	34.65	33.59	32.41	31.46	31.89	32.63	32.38	32.84	31.81
B04	40	34.60	40	40	36.92	40	35.05	40	40	40	40	33.58
B05	34.80	33.22	34.10	33.26	30.81	32.57	32.66	35.59	32.79	33.09	30.72	32.94
B06	33.99	34.21	33.55	34.55	33.68	33.14	32.85	33.28	32.68	33.28	33.19	32.84
B07	32.49	32.86	31.82	32.85	32.29	31.36	30.37	31.93	31.26	32.18	33.77	32.77
B08	29.00	29.61	28.18	29.80	30.20	29.58	26.61	28.27	27.60	29.39	27.14	27.17
B09	35.71	35.74	35.50	34.23	34.48	32.28	34.46	33.74	33.73	33.68	33.67	35.51
B10	32.59	31.98	32.54	31.75	32.06	31.27	31.28	32.28	31.50	31.46	32.41	32.49
B11	29.36	29.38	29.13	29.48	28.88	27.94	28.87	29.16	29.66	30.49	29.48	29.47
B12	27.58	26.34	25.37	26.64	26.29	25.26	25.02	24.98	25.14	25.17	25.30	23.84

C01	30.04	29.61	30.59	27.91	29.20	30.54	28.20	29.29	29.47	28.85	29.24	30.28
C02	31.53	32.41	31.33	31.79	32.19	29.95	30.91	32.20	31.73	31.95	31.89	30.53
C03	28.92	29.56	28.97	28.84	30.63	28.12	27.62	29.09	28.18	28.60	29.43	28.72
C04	26.25	29.59	27.53	28.44	29.79	24.64	22.66	27.25	25.51	23.06	27.57	23.45
C05	32.76	32.11	30.85	33.47	29.10	30.00	29.92	30.96	30.65	29.99	30.05	29.40
C06	36.42	37.32	36.16	34.73	35.41	33.67	36.53	36.50	35.35	36.69	36.15	34.88
C07	23.23	23.75	23.00	24.51	24.79	23.41	23.40	22.57	22.85	21.34	22.92	21.99
C08	29.72	29.75	28.96	30.60	29.80	29.50	27.66	28.12	27.86	26.47	27.91	27.49
C09	37.66	36.07	34.16	35.91	33.78	33.03	33.14	34.25	33.88	35.90	33.66	33.99
C10	27.44	28.00	24.67	25.93	26.00	23.51	24.99	24.43	24.28	27.04	24.57	22.52
C11	33.62	34.44	34.09	34.08	32.30	32.65	32.65	32.99	32.92	33.74	33.19	31.47
C12	31.79	32.27	31.26	31.50	30.75	30.50	30.75	31.32	30.51	29.62	31.00	30.59
D01	31.68	31.65	32.16	31.35	30.74	31.38	30.01	30.38	30.87	30.00	30.91	33.19
D02	23.28	23.57	23.22	22.99	22.27	20.86	21.10	22.92	21.31	20.93	22.88	21.18
D03	35.13	36.09	35.42	36.60	36.51	33.96	34.41	36.34	36.32	35.51	36.41	35.33
D04	32.53	33.45	34.08	35.35	32.99	32.45	34.43	34.22	33.17	34.60	34.72	34.32
D05	32.18	30.45	29.97	34.77	26.93	30.70	31.59	32.13	32.59	31.38	26.25	31.50
D06	27.90	27.21	26.23	29.10	29.20	27.85	25.57	27.13	27.48	25.22	26.82	25.84
D07	32.28	32.23	30.51	31.56	30.61	29.09	29.99	32.27	31.39	32.72	31.94	30.47
D08	32.98	32.71	33.84	33.35	33.23	31.62	32.70	33.10	32.51	32.17	33.41	31.25
D09	29.46	30.85	30.76	30.34	29.78	29.09	30.62	30.54	29.22	29.01	29.85	30.78
D10	28.45	28.34	27.72	28.88	28.28	27.96	27.22	26.89	27.50	27.74	26.87	26.42
D11	33.14	31.93	31.33	33.58	33.68	31.74	32.69	31.84	31.25	33.41	33.81	29.95
D12	36.91	35.22	35.38	36.81	34.49	34.49	34.62	38.03	36.07	37.01	33.77	34.46
E01	30.27	30.25	30.95	30.19	30.12	29.52	29.60	29.41	29.69	30.14	30.27	30.19
E02	29.96	31.01	31.34	32.18	31.24	29.98	30.14	31.57	29.35	29.50	30.67	30.09
E03	31.12	32.52	32.37	33.02	31.78	29.82	29.96	31.31	31.20	31.11	31.97	30.64
E04	27.69	30.92	29.21	32.43	32.85	30.84	27.44	30.11	30.28	28.57	30.95	29.31
E05	29.86	28.82	28.16	29.63	30.24	27.56	25.96	28.20	27.74	26.42	28.38	26.85
E06	35.88	35.56	35.73	34.64	33.93	33.78	34.00	33.90	34.06	33.84	34.23	34.88
E07	24.00	25.26	25.19	26.98	26.51	26.55	26.81	25.98	25.29	26.63	26.51	26.44
E08	28.36	28.79	28.92	28.78	29.60	28.96	25.99	29.69	28.61	29.86	29.95	28.73
E09	29.40	30.66	29.45	33.18	32.12	30.32	28.69	31.15	30.55	31.48	31.68	30.61
E10	30.00	29.53	28.33	29.57	29.35	27.64	27.99	28.23	27.91	28.82	27.55	25.94

E11	31.80	31.83	31.39	32.06	31.32	29.78	30.74	31.02	30.13	31.27	31.38	30.59
E12	34.25	29.91	32.65	33.99	28.34	30.18	32.31	27.56	32.11	33.62	25.98	31.14
F01	29.45	30.53	30.19	29.00	31.35	30.66	29.92	30.81	30.07	29.87	30.83	30.27
F02	32.76	34.80	34.81	32.64	34.19	33.44	32.34	33.41	33.10	32.41	34.69	34.51
F03	23.57	23.94	23.02	23.71	23.80	22.13	19.99	21.60	21.41	19.59	21.01	19.87
F04	32.75	34.61	35.17	33.74	34.10	34.61	33.92	36.58	33.90	34.04	36.51	33.50
F05	24.58	24.33	22.83	23.90	24.71	22.33	20.48	23.06	22.69	20.56	23.09	20.86
F06	35.65	36.91	35.37	37.16	36.05	34.95	34.30	38.13	36.37	34.37	37.45	35.98
F07	29.33	28.44	26.67	29.57	28.78	26.71	24.88	27.65	27.37	28.76	26.97	26.00
F08	32.08	33.27	32.46	34.49	31.77	31.00	32.72	32.45	31.69	31.99	32.42	31.32
F09	32.38	32.49	31.81	32.09	32.39	30.21	31.85	32.58	31.14	32.33	32.77	31.46
F10	22.40	21.56	20.25	20.82	21.02	19.83	21.19	21.57	20.50	20.57	19.90	19.57
F11	32.24	32.78	31.74	31.92	32.14	29.95	31.90	32.32	31.05	31.55	32.29	31.74
F12	33.28	33.83	31.82	33.39	32.83	30.64	31.70	32.14	31.68	34.11	33.56	30.82
G01	32.62	32.32	33.60	33.20	33.76	31.20	31.31	30.99	31.82	31.84	31.19	33.61
G02	30.70	32.92	31.90	34.04	33.29	32.93	32.93	31.32	34.35	30.91	29.23	30.97
G03	25.93	26.61	25.82	27.17	27.15	25.97	24.33	25.92	25.89	27.63	27.35	26.62
G04	26.16	26.86	26.10	25.75	25.46	23.84	23.96	25.50	26.00	24.15	26.02	24.90
G05	30.20	31.47	30.63	31.25	30.85	29.55	29.82	30.83	30.61	29.37	31.17	29.14
G06	40	35.58	34.07	10.58	34.42	40	35.52	40	38.63	33.45	32.87	33.30
G07	28.38	29.40	26.67	28.71	31.66	25.89	22.30	27.14	26.61	25.96	26.76	24.13
G08	31.23	30.44	30.00	29.30	29.52	28.40	28.29	30.82	29.78	29.46	30.85	30.11
G09	32.88	31.61	31.41	31.34	31.28	29.63	30.51	31.95	31.44	31.72	32.50	31.33
G10	28.63	27.84	27.61	29.38	28.89	27.88	27.64	27.17	27.62	28.26	27.31	26.59
G11	24.36	24.46	23.44	25.53	23.13	22.22	24.36	24.19	23.83	24.19	23.21	22.33
G12	35.76	35.57	35.57	37.72	31.79	31.78	37.69	32.51	36.57	38.26	31.15	32.88
H01	20.61	23.63	20.89	21.00	22.56	17.22	18.59	22.03	19.25	19.56	22.22	19.52
H02	20.60	22.05	20.97	21.73	21.13	20.09	17.46	20.20	18.46	18.33	20.24	18.79
H03	20.37	22.68	20.18	21.63	19.69	19.69	19.13	19.86	21.15	19.50	19.79	19.50
H04	26.30	26.68	25.93	26.23	25.80	25.16	24.96	26.13	26.35	25.25	26.14	25.32
H05	22.68	21.40	20.23	19.92	19.64	18.31	16.57	19.10	20.49	17.44	19.49	17.63
H06	35.22	35.67	36.41	34.31	34.93	34.44	35.26	35.75	35.50	35.17	35.68	34.62
H07	22.40	23.64	23.88	23.71	25.29	23.19	21.12	25.39	26.30	21.13	24.90	22.89
H08	22.50	23.83	24.00	24.51	25.68	22.81	21.40	25.11	25.96	21.22	24.80	22.732

H09	22.59	23.77	23.65	24.38	25.36	22.84	25.84	24.87	23.68	26.77	25.12	22.97
H10	27.66	23.23	27.91	27.13	23.89	25.71	28.59	23.52	26.43	28.30	22.87	24.21
H11	28.17	23.90	27.31	28.16	22.66	23.92	29.17	24.15	25.87	27.64	22.27	23.71
H12	28.80	22.92	27.84	27.73	23.81	24.25	29.33	23.82	26.68	28.15	22.17	23.90

Disease Models & Mechanisms • Supplementary information

Table S3. Fold regulation and p-values of genes associated mucosal inflammation. Fold regulation is compared to controls

		Group 1		Group 2		Group 3	
**Position**	**Gene Symbol**	**Fold Regulation**	**p-Value**	**Fold Regulation**	**p-Value**	**Fold Regulation**	**p-Value**
A01	**ADIPOQ**	-1.34	0.949612	-2.65	0.07756	-4.34	0.022261
A02	**BMP2**	-2.93	0.162542	-2.56	0.214097	-3.07	0.150726
A03	**BMP4**	2.89	0.048146	-1.93	0.019491	-2.22	0.007207
A04	**BMP6**	-1.13	0.333216	-1.62	0.024908	-1.6	0.015384
A05	**BMP7**	2.49	0.044874	-2.19	0.15756	-5.99	0.051444
A06	**C5**	-2.99	0.26999	1.42	0.521938	2.12	0.946686
A07	**CCL1**	2.87	0.296137	2.57	0.391479	2.56	0.40323
A08	**CCL11**	-2.17	0.211494	2.93	0.009991	2.97	0.003055
A09	**CCL13**	1.06	0.855139	-1.1	0.634625	-1	0.89173
A10	**CCL17**	-1.27	0.847791	-3.24	0.228373	-3.95	0.188997
A11	**CCL18**	-1.03	0.717673	-1.94	0.633077	-4.78	0.471747
A12	**CCL19**	-1.08	0.653829	-2.16	0.832994	-2.47	0.749384
B01	**CCL2**	1.93	0.005131	1.01	0.765233	-1.59	0.049993
B02	**CCL20**	-1.49	0.161355	-1.85	0.180155	-1.1	0.733896
B03	**CCL21**	-3.81	0.076845	-2.06	0.224412	-2.76	0.11197
B04	**CCL22**	-2.23	0.29771	-3.56	0.192872	-2.69	0.229169
B05	**CCL24**	1.74	0.560675	-2.2	0.38765	1.02	0.733473
B06	**CCL3**	-1.87	0.158638	-1.65	0.368825	-1.93	0.216966
B07	**CCL5**	-1.75	0.090679	-1.41	0.254146	-4.87	0.010946
B08	**CCL7**	-3.88	0.008	-1.2	0.425252	-1.66	0.946204
B09	**CCL8**	1.24	0.778056	-1.6	0.610014	-1.85	0.510117
B10	**CD40LG**	-1.28	0.57854	-2.02	0.329164	-2.86	0.238628
B11	**CNTF**	-1.41	0.364815	-3.11	0.121358	-4.88	0.090452
B12	**CSF1**	-1.57	0.295875	-1.24	0.661535	-1.08	0.72296
C01	**CSF2**	-1.12	0.769752	-1.52	0.413178	-2.21	0.358839
C02	**CSF3**	-1.5	0.0961	-2.94	0.007822	-2.76	0.021024
C03	**CX3CL1**	-2.11	0.219691	-1.8	0.153513	-2.89	0.038237
C04	**CXCL1**	-1.82	0.646625	1.93	0.39189	2.51	0.224539

Disease Models & Mechanisms • Supplementary information

C05	**CXCL10**	1.02	0.615737	-1.23	0.522227	1.25	0.685628
C06	**CXCL11**	-1.41	0.413821	-3.25	0.14523	-3.31	0.134927
C07	**CXCL12**	-3.83	0.02162	-2.49	0.18512	-1.44	0.200292
C08	**CXCL13**	-2.86	0.074479	-1.07	0.980486	1.34	0.426084
C09	**CXCL16**	-1.18	0.626293	-1.67	0.221699	-2.4	0.319296
C10	**CXCL2**	1.44	0.761869	1.35	0.610594	1.17	0.492224
C11	**CXCL5**	1.01	0.81883	-1.42	0.487189	-1.43	0.658705
C12	**CXCL9**	-1.13	0.838286	-1.72	0.288454	-1.31	0.369544
D01	**FASLG**	-1.28	0.71466	-1.22	0.660594	-2.46	0.386366
D02	**GPI**	1.22	0.645905	-1.08	0.946249	-1.05	0.721945
D03	**IFNA2**	-1.6	0.316888	-2.84	0.149693	-3.4	0.133243
D04	**IFNG**	-2.22	0.203707	-4.87	0.106166	-7.76	0.068464
D05	**IL10**	-1.95	0.562303	-7.67	0.137885	-1.53	0.501363
D06	**IL11**	-6.18	0.062115	-2.48	0.13037	-1.53	0.280914
D07	**IL12A**	1.17	0.723315	-2.36	0.071131	-3.48	0.071281
D08	**IL12B**	-1.5	0.380721	-2.45	0.284113	-1.82	0.332568
D09	**IL13**	-1.33	0.399013	-2.76	0.212357	-2.44	0.181454
D10	**IL15**	-2.34	0.136928	-1.66	0.493474	-1.52	0.603639
D11	**IL16**	-3.72	0.139453	-2.81	0.272895	-4.06	0.244233
D12	**IL17A**	-1.61	0.392563	-2.97	0.147803	-2.26	0.414697
E01	**IL17F**	-1.4	0.462483	-1.71	0.43161	-2.77	0.209698
E02	**IL18**	-2.62	0.111179	-2.43	0.273233	-2.11	0.153393
E03	**IL1A**	-1.47	0.328236	-1.43	0.303056	-2	0.144028
E04	**IL1B**	-13.84	0.135316	-3.26	0.214877	-4.29	0.184107
E05	**IL1RN**	-2.34	0.19862	-1.04	0.648971	-1.03	0.673274
E06	**IL2**	-1.11	0.78615	-1.61	0.473853	-2.12	0.285518
E07	**IL21**	-7.39	0.041969	-7.5	0.050909	-11.12	0.035929
E08	**IL22**	-2.73	0.169701	-2.15	0.199245	-6.02	0.062789
E09	**IL23A**	-8.35	0.001218	-3.98	0.002164	-9.1	0.001272
E10	**IL24**	-1.51	0.238463	-1.37	0.578921	1.06	0.614469
E11	**IL27**	-1.33	0.380075	-1.57	0.533096	-2.25	0.15919
E12	**IL3**	1.33	0.95802	-1.06	0.815109	1.2	0.484133
F01	**IL4**	-2.48	0.566347	-3.77	0.046445	-4.1	0.035901

Disease Models & Mechanisms • Supplementary information

F02	**IL5**	-1.26	0.866017	-1.44	0.388842	-2.85	0.189438
F03	**IL6**	-1.66	0.108928	1.75	0.049729	3.01	0.001628
F04	**IL7**	-2.08	0.337616	-3.61	0.146737	-3.55	0.134236
F05	**CXCL8**	-1.69	0.20717	1.1	0.942692	1.57	0.262679
F06	**IL9**	-2.01	0.281961	-2.77	0.1509	-2.94	0.14391
F07	**LIF**	-2.35	0.144571	-1.14	0.521211	-1.82	0.501239
F08	**LTA**	-1.79	0.352771	-2.61	0.128913	-2.1	0.06198
F09	**LTB**	-1.28	0.403547	-2.51	0.184707	-3.31	0.055584
F10	**MIF**	-1.13	0.592163	-2.6	0.184788	-1.3	0.683908
F11	**MSTN**	-1.08	0.686789	-2.3	0.180819	-2.59	0.024663
F12	**NODAL**	-1.26	0.392322	-1.47	0.451274	-3.06	0.1661
G01	**OSM**	-1.87	0.323128	-1.17	0.740635	-2.19	0.552686
G02	**PPBP**	-6.1	0.0713	-6.65	0.144067	-1.23	0.78631
G03	**SPP1**	-3.18	0.019935	-1.94	0.054915	-7.17	0.009913
G04	**TGFB2**	1.26	0.291319	-1.39	0.263056	-1.33	0.261376
G05	**THPO**	-1.75	0.070771	-2.54	0.037897	-1.85	0.06717
G06	**TNF**	127.49	0.373901	-4.02	0.050591	-1.22	0.969815
G07	**TNFRSF11B**	-3.09	0.268953	2.14	0.359668	1.7	0.344565
G08	**TNFSF10**	1.37	0.659453	-1.71	0.292383	-2.54	0.176298
G09	**TNFSF11**	1.14	0.847909	-2.05	0.280084	-3.14	0.196984
G10	**TNFSF13B**	-3.29	0.158789	-2.22	0.331585	-2.18	0.332172
G11	**VEGFA**	-1.48	0.522086	-3.33	0.065147	-1.89	0.334883
G12	**XCL1**	2.17	0.328343	-1.83	0.947324	1.17	0.480846
H01	**ACTB**	1.34	0.498357	1.04	0.823407	-1.4	0.424168
H02	**B2M**	-1.74	0.069622	1.75	0.198236	1.25	0.304749
H03	**GAPDH**	-1.22	0.842034	-1.59	0.393738	-1.22	0.668975
H04	**HPRT1**	-1.37	0.36067	-2.31	0.067216	-2.04	0.08229
H05	**RPLP0**	2.17	0.136325	2.02	0.292426	2.79	0.046283

Table S4. Table of genes involved in the Fatty Acid Metabolism (PAHS-007Z).

Position	RefSeq Number	Symbol	Description
A01	NM_001607	**ACAA1**	Acetyl-CoA acyltransferase 1
A02	NM_006111	**ACAA2**	Acetyl-CoA acyltransferase 2
A03	NM_025247	**ACAD10**	Acyl-CoA dehydrogenase family, member 10
A04	NM_032169	**ACAD11**	Acyl-CoA dehydrogenase family, member 11
A05	NM_014049	**ACAD9**	Acyl-CoA dehydrogenase family, member 9
A06	NM_001608	**ACADL**	Acyl-CoA dehydrogenase, long chain
A07	NM_000016	**ACADM**	Acyl-CoA dehydrogenase, C-4 to C-12 straight chain
A08	NM_000017	**ACADS**	Acyl-CoA dehydrogenase, C-2 to C-3 short chain
A09	NM_001609	**ACADSB**	Acyl-CoA dehydrogenase, short/branched chain
A10	NM_000018	**ACADVL**	Acyl-CoA dehydrogenase, very long chain
A11	NM_000019	**ACAT1**	Acetyl-CoA acetyltransferase 1
A12	NM_005891	**ACAT2**	Acetyl-CoA acetyltransferase 2
B01	NM_001037161	**ACOT1**	Acyl-CoA thioesterase 1
B02	NM_130767	**ACOT12**	Acyl-CoA thioesterase 12
B03	NM_001037162	**ACOT6**	Acyl-CoA thioesterase 6
B04	NM_181866	**ACOT7**	Acyl-CoA thioesterase 7
B05	NM_005469	**ACOT8**	Acyl-CoA thioesterase 8
B06	NM_001033583	**ACOT9**	Acyl-CoA thioesterase 9
B07	NM_004035	**ACOX1**	Acyl-CoA oxidase 1, palmitoyl
B08	NM_003500	**ACOX2**	Acyl-CoA oxidase 2, branched chain
B09	NM_003501	**ACOX3**	Acyl-CoA oxidase 3, pristanoyl
B10	NM_015162	**ACSBG1**	Acyl-CoA synthetase bubblegum family member 1
B11	NM_030924	**ACSBG2**	Acyl-CoA synthetase bubblegum family member 2
B12	NM_001995	**ACSL1**	Acyl-CoA synthetase long-chain family member 1
C01	NM_004457	**ACSL3**	Acyl-CoA synthetase long-chain family member 3
C02	NM_004458	**ACSL4**	Acyl-CoA synthetase long-chain family member 4

Disease Models & Mechanisms • Supplementary information

C03	NM_016234	**ACSL5**	Acyl-CoA synthetase long-chain family member 5
C04	NM_001009185	**ACSL6**	Acyl-CoA synthetase long-chain family member 6
C05	NM_005622	**ACSM3**	Acyl-CoA synthetase medium-chain family member 3
C06	NM_001080454	**ACSM4**	Acyl-CoA synthetase medium-chain family member 4
C07	NM_017888	**ACSM5**	Acyl-CoA synthetase medium-chain family member 5
C08	NM_000690	**ALDH2**	Aldehyde dehydrogenase 2 family (mitochondrial)
C09	NM_004051	**BDH1**	3-hydroxybutyrate dehydrogenase, type 1
C10	NM_020139	**BDH2**	3-hydroxybutyrate dehydrogenase, type 2
C11	NM_001876	**CPT1A**	Carnitine palmitoyltransferase 1A (liver)
C12	NM_004377	**CPT1B**	Carnitine palmitoyltransferase 1B (muscle)
D01	NM_152359	**CPT1C**	Carnitine palmitoyltransferase 1C
D02	NM_000098	**CPT2**	Carnitine palmitoyltransferase 2
D03	NM_000755	**CRAT**	Carnitine O-acetyltransferase
D04	NM_021151	**CROT**	Carnitine O-octanoyltransferase
D05	NM_001359	**DECR1**	2,4-dienoyl CoA reductase 1, mitochondrial
D06	NM_020664	**DECR2**	2,4-dienoyl CoA reductase 2, peroxisomal
D07	NM_004092	**ECHS1**	Enoyl CoA hydratase, short chain, 1, mitochondrial
D08	NM_006117	**ECI2**	Enoyl-CoA delta isomerase 2
D09	NM_001966	**EHHADH**	Enoyl-CoA, hydratase/3-hydroxyacyl CoA dehydrogenase
D10	NM_001443	**FABP1**	Fatty acid binding protein 1, liver
D11	NM_000134	**FABP2**	Fatty acid binding protein 2, intestinal
D12	NM_004102	**FABP3**	Fatty acid binding protein 3, muscle and heart (mammary-derived growth inhibitor)
E01	NM_001442	**FABP4**	Fatty acid binding protein 4, adipocyte
E02	NM_001444	**FABP5**	Fatty acid binding protein 5 (psoriasis-associated)
E03	NM_001445	**FABP6**	Fatty acid binding protein 6, ileal
E04	NM_001446	**FABP7**	Fatty acid binding protein 7, brain
E05	NM_004104	**FASN**	Fatty acid synthase
E06	NM_000159	**GCDH**	Glutaryl-CoA dehydrogenase

Disease Models & Mechanisms • Supplementary information

E07	NM_000167	**GK**	Glycerol kinase
E08	NM_033214	**GK2**	Glycerol kinase 2
E09	NM_005276	**GPD1**	Glycerol-3-phosphate dehydrogenase 1 (soluble)
E10	NM_000408	**GPD2**	Glycerol-3-phosphate dehydrogenase 2 (mitochondrial)
E11	NM_000182	**HADHA**	Hydroxyacyl-CoA dehydrogenase/3-ketoacyl-CoA thiolase/enoyl-CoA hydratase (trifunctional protein), alpha subunit
E12	NM_000191	**HMGCL**	3-hydroxymethyl-3-methylglutaryl-CoA lyase
F01	NM_002130	**HMGCS1**	3-hydroxy-3-methylglutaryl-CoA synthase 1 (soluble)
F02	NM_005518	**HMGCS2**	3-hydroxy-3-methylglutaryl-CoA synthase 2 (mitochondrial)
F03	NM_005357	**LIPE**	Lipase, hormone-sensitive
F04	NM_000237	**LPL**	Lipoprotein lipase
F05	NM_032601	**MCEE**	Methylmalonyl CoA epimerase
F06	NM_000255	**MUT**	Methylmalonyl CoA mutase
F07	NM_022120	**OXCT2**	3-oxoacid CoA transferase 2
F08	NM_018441	**PECR**	Peroxisomal trans-2-enoyl-CoA reductase
F09	NM_021129	**PPA1**	Pyrophosphatase (inorganic) 1
F10	NM_006251	**PRKAA1**	Protein kinase, AMP-activated, alpha 1 catalytic subunit
F11	NM_006252	**PRKAA2**	Protein kinase, AMP-activated, alpha 2 catalytic subunit
F12	NM_006253	**PRKAB1**	Protein kinase, AMP-activated, beta 1 non-catalytic subunit
G01	NM_005399	**PRKAB2**	Protein kinase, AMP-activated, beta 2 non-catalytic subunit
G02	NM_002730	**PRKACA**	Protein kinase, cAMP-dependent, catalytic, alpha
G03	NM_182948	**PRKACB**	Protein kinase, cAMP-dependent, catalytic, beta
G04	NM_002733	**PRKAG1**	Protein kinase, AMP-activated, gamma 1 non-catalytic subunit
G05	NM_016203	**PRKAG2**	Protein kinase, AMP-activated, gamma 2 non-catalytic subunit
G06	NM_017431	**PRKAG3**	Protein kinase, AMP-activated, gamma 3 non-catalytic subunit
G07	NM_198580	**SLC27A1**	Solute carrier family 27 (fatty acid transporter), member 1
G08	NM_003645	**SLC27A2**	Solute carrier family 27 (fatty acid transporter), member 2
G09	NM_024330	**SLC27A3**	Solute carrier family 27 (fatty acid transporter), member 3

G10	NM_005094	**SLC27A4**	Solute carrier family 27 (fatty acid transporter), member 4
G11	NM_012254	**SLC27A5**	Solute carrier family 27 (fatty acid transporter), member 5
G12	NM_014031	**SLC27A6**	Solute carrier family 27 (fatty acid transporter), member 6
H01	NM_001101	**ACTB**	Actin, beta
H02	NM_004048	**B2M**	Beta-2-microglobulin
H03	NM_002046	**GAPDH**	Glyceraldehyde-3-phosphate dehydrogenase
H04	NM_000194	**HPRT1**	Hypoxanthine phosphoribosyltransferase 1
H05	NM_001002	**RPLP0**	Ribosomal protein, large, P0
H06	SA_00105	**HGDC**	Human Genomic DNA Contamination
H07	SA_00104	**RTC**	Reverse Transcription Control
H08	SA_00104	**RTC**	Reverse Transcription Control
H09	SA_00104	**RTC**	Reverse Transcription Control
H10	SA_00103	**PPC**	Positive PCR Control
H11	SA_00103	**PPC**	Positive PCR Control
H12	SA_00103	**PPC**	Positive PCR Control

Table S5. List of CT values of genes involved in fatty acid metabolism that was uploaded on to the data analysis web portal at http://www.qiagen.com/geneglobe

Position	Control Group	Control Group	Control Group	PG/VG Test Group 1	PG/VG Test Group 1	PG/VG Test Group 1	PG/VG+N Test Group 2	PG/VG+N Test Group 2	PG/VG+N Test Group 2	PG/VG+N+F Test Group 3	PG/VG+N+F Test Group 3	PG/VG+N+F Test Group 3
A01	25.455	24.70	26.12	25.91	25.70	25.38	25.24	25.12	25.79	25.82	25.69	25.68
A02	24.443	25.43	24.87	23.59	24.43	24.53	23.21	23.33	23.75	23.45	23.69	24.34
A03	26.68	36.24	26.78	25.67	26.24	26.21	25.07	24.84	25.64	25.71	25.62	25.53
A04	25.21	27.57	25.77	24.94	24.57	24.99	24.46	24.63	24.86	24.84	24.89	25.20
A05	26.45	22.39	26.23	25.83	25.89	25.63	25.29	25.33	25.54	25.71	25.70	25.79
A06	38.00	26.35	33.81	40	33.85	36.47	30.53	29.66	29.64	31.18	30.24	30.75
A07	40	35.86	38.31	34.61	35.86	32.92	28.13	28.43	28.71	30.29	31.16	31.21
A08	27.35	27.25	26.89	26.83	27.25	26.57	26.28	26.39	26.51	27.06	27.12	27.18
A09	27.27	26.50	26.78	26.31	26.50	25.87	25.71	25.84	25.70	26.59	26.46	26.71
A10	22.31	22.24	22.35	22.54	22.24	21.60	20.92	21.09	21.73	21.58	22.23	22.42
A11	25.74	24.72	25.65	25.09	24.72	24.94	23.77	23.86	25.11	24.76	25.12	25.37
A12	26.31	25.62	26.35	25.28	25.62	25.02	24.75	25.14	25.25	26.16	25.21	25.57
B01	32.18	23.13	31.57	31.27	31.13	31.29	31.20	31.14	31.20	31.64	30.71	30.95
B02	30.62	36.61	31.78	32.62	30.61	31.23	30.42	29.57	30.63	31.54	32.28	31.17
B03	34.80	32.33	35.21	39.10	34.33	34.29	34.38	34.94	33.89	34.54	34.07	33.65
B04	27.04	26.73	26.93	25.17	25.43	25.45	26.15	25.73	25.78	25.98	26.31	25.68
B05	27.12	24.91	27.29	25.94	26.91	27.08	26.00	26.53	26.54	26.32	26.42	26.71
B06	26.21	31.34	26.52	24.96	25.34	25.40	24.26	24.40	25.14	25.65	25.59	25.64
B07	26.27	25.67	26.44	25.18	25.67	25.23	24.86	24.76	25.00	25.81	25.71	26.36
B08	31.36	31.43	30.87	30.72	31.43	30.47	29.37	29.32	30.21	29.63	31.45	31.15
B09	27.56	27.19	27.76	27.34	27.19	26.94	26.93	27.16	26.68	27.89	27.44	27.38
B10	30.81	31.54	31.26	29.84	31.54	30.21	28.17	30.06	29.93	29.20	30.97	30.91
B11	27.33	27.52	28.02	27.79	27.52	27.75	28.29	28.67	28.59	28.22	28.94	28.70
B12	25.64	24.51	25.52	26.10	24.51	24.44	25.17	25.72	22.94	25.98	24.35	24.54
C01	25.35	23.74	26.25	26.12	26.74	25.46	24.14	24.76	23.95	25.86	25.55	25.07
C02	23.00	30.74	24.13	23.78	23.74	23.45	22.30	22.73	23.41	22.62	23.66	25.08
C03	28.60	28.10	28.51	31.34	29.10	28.96	28.52	28.52	28.49	28.24	28.11	28.58
C04	37.39	35.16	35.92	33.68	34.16	33.97	33.80	33.19	32.97	32.65	30.94	30.90

Disease Models & Mechanisms • Supplementary information

C05	28.58	24.69	28.61	28.97	28.69	28.31	28.31	27.99	27.51	28.45	27.95	28.58
C06	32.67	26.15	31.90	32.88	32.15	32.27	31.52	31.92	30.34	32.18	32.38	31.45
C07	29.02	29.30	29.03	29.47	29.30	29.11	29.65	29.60	29.07	30.03	29.75	29.90
C08	26.72	28.24	26.82	26.24	28.24	26.53	25.47	25.69	26.38	27.34	27.21	26.84
C09	30.86	30.85	31.23	30.35	30.85	30.48	30.15	29.51	28.22	30.92	30.39	30.01
C10	24.14	24.88	24.21	24.34	24.88	24.92	22.89	23.48	24.28	23.98	23.95	24.19
C11	26.46	26.12	26.82	26.50	26.12	26.07	24.58	24.78	26.63	25.24	26.34	26.50
C12	31.19	31.31	31.74	31.70	31.31	31.24	31.28	31.29	31.79	31.92	31.18	31.39
D01	25.49	26.92	25.57	25.21	26.92	26.15	24.60	24.66	25.39	25.50	26.25	25.77
D02	27.50	24.94	26.74	27.73	26.94	27.15	27.82	27.27	26.55	28.28	27.65	27.46
D03	26.30	32.19	25.84	24.74	25.19	24.87	24.58	24.58	24.24	24.56	24.84	24.53
D04	26.87	33.25	27.29	25.95	26.25	25.86	24.63	25.2	25.35	24.94	25.79	25.43
D05	24.27	29.44	24.92	23.90	24.44	23.25	22.41	23.10	24.36	22.71	24.84	24.89
D06	26.40	28.96	26.08	26.42	25.96	25.79	26.91	26.72	26.32	26.55	26.47	25.98
D07	24.70	24.40	24.79	23.19	24.40	23.72	23.65	23.47	22.84	24.33	24.60	24.53
D08	25.23	25.45	25.60	24.03	25.45	25.05	23.71	24.40	24.34	24.35	25.65	25.18
D09	29.29	30.39	29.75	29.49	30.39	29.53	27.24	28.26	28.18	28.26	29.52	29.25
D10	26.55	31.43	26.97	31.24	31.43	30.88	31.53	32.19	31.18	29.81	29.76	29.34
D11	33.23	34.21	32.88	34.34	34.21	34.66	33.30	34.45	39.65	32.99	37.15	36.84
D12	26.72	26.76	26.65	25.62	26.76	26.46	26.16	26.33	23.96	26.65	24.86	24.99
E01	28.90	28.43	29.36	28.72	28.43	28.67	26.64	26.68	30.76	27.21	29.90	29.50
E02	23.80	21.65	24.66	23.51	21.65	20.86	22.52	23.71	22.65	23.87	22.79	23.31
E03	30.63	29.81	29.42	30.48	29.81	29.95	30.73	30.27	29.73	30.14	29.65	29.87
E04	31.15	30.58	30.87	30.66	30.58	30.81	30.88	31.18	31.57	30.93	31.33	31.19
E05	24.93	23.67	25.37	24.31	23.67	23.32	24.12	24.09	23.46	24.41	24.40	25.28
E06	28.01	27.85	28.42	26.92	27.85	27.67	26.98	26.94	27.24	27.86	27.50	27.61
E07	34.91	34.42	34.39	35.91	34.42	32.45	31.41	32.78	32.95	32.97	34.74	34.60
E08	37.22	38.27	34.08	36.13	38.27	35.90	37.19	36.96	38.90	37.23	40	40
E09	31.69	35.65	33.30	29.17	35.65	32.66	31.95	31.85	31.77	33.41	32.98	33.72
E10	24.65	25.41	25.12	25.11	25.41	24.66	23.67	24.18	24.46	24.36	24.72	24.78
E11	24.60	24.39	24.67	23.84	24.39	24.21	23.28	23.73	23.78	24.17	24.33	24.35
E12	25.65	25.58	26.09	24.63	25.58	25.19	24.77	24.63	23.87	25.82	24.61	24.56
F01	22.74	23.07	23.40	23.15	23.07	22.69	22.78	22.80	21.90	24.10	22.56	22.33
F02	31.29	37.48	32.83	34.32	37.48	32.87	33.93	39.06	32.88	40	34.59	32.95
F03	27.67	27.73	27.39	27.60	27.73	27.43	27.59	27.33	27.25	27.74	27.74	27.66
F04	28.82	28.34	28.94	25.40	28.34	27.84	25.94	26.27	26.30	25.60	27.25	27.17

F05	28.00	27.52	28.24	27.52	27.52	27.18	26.05	26.52	26.53	26.41	27.47	27.57
F06	26.04	26.37	26.72	25.95	26.37	25.94	24.96	25.31	25.51	25.02	25.64	26.22
F07	30.48	30.67	29.12	29.74	30.67	29.58	28.59	28.44	29.25	29.23	29.42	30.28
F08	28.72	27.95	28.81	26.36	27.95	27.10	26.15	26.40	27.03	27.23	27.29	27.40
F09	23.92	24.18	24.39	22.94	24.18	23.58	22.42	23.06	22.99	23.36	23.81	23.58
F10	25.09	25.39	25.65	24.71	25.39	23.96	23.24	23.77	23.97	24.41	24.35	24.56
F11	28.49	30.09	29.92	27.47	30.09	28.71	27.56	28.34	28.08	28.54	29.33	28.91
F12	27.95	28.12	28.48	25.70	28.12	27.43	26.22	26.78	25.66	27.14	26.99	26.96
G01	26.45	27.10	27.46	26.66	27.10	27.14	25.61	26.20	25.39	25.87	26.00	26.20
G02	26.19	27.00	27.36	26.64	27.00	26.42	25.01	25.38	24.51	25.20	25.78	25.60
G03	26.70	25.88	26.08	24.79	25.88	25.79	24.62	24.93	24.72	24.70	25.17	24.86
G04	25.00	24.77	25.69	24.14	24.77	24.77	23.54	23.45	24.21	23.59	24.69	24.19
G05	27.61	27.37	27.62	26.18	27.37	27.10	25.87	26.39	25.95	26.42	26.56	26.98
G06	32.70	33.50	31.90	33.58	33.50	31.60	37.20	34.93	28.48	33.06	29.17	30.71
G07	28.39	28.21	28.16	27.42	28.21	27.75	27.50	27.64	26.91	28.42	27.85	28.51
G08	33.38	34.21	32.91	32.37	34.21	32.46	32.49	32.63	29.28	32.52	33.60	32.28
G09	28.50	28.88	28.97	27.31	28.88	28.08	27.85	27.51	27.08	28.44	28.29	28.07
G10	29.39	29.21	29.94	27.71	29.21	28.17	27.71	27.62	27.89	29.36	29.22	28.81
G11	28.11	27.69	27.75	28.07	27.69	27.41	28.32	28.24	27.94	28.55	27.59	27.87
G12	28.99	31.94	29.81	30.11	31.94	29.10	26.79	27.63	28.52	29.25	29.53	29.21
H01	19.61	19.82	20.21	18.52	19.82	18.97	18.95	18.88	17.82	19.50	19.18	18.97
H02	19.64	20.37	20.91	20.00	20.37	19.77	17.83	18.73	19.77	18.20	19.52	20.07
H03	20.97	19.78	20.17	19.61	19.78	19.19	20.01	19.23	17.95	20.16	20.12	19.39
H04	26.00	25.64	26.24	24.58	25.64	25.95	24.65	24.40	24.94	24.77	25.75	25.35
H05	20.42	19.06	20.44	18.15	19.06	18.37	18.23	17.30	17.63	17.80	18.91	18.96
H06	35.81	36.13	35.70	36.98	36.13	34.09	38.56	33.56	36.59	34.50	35.38	35.69
H07	21.89	23.77	23.15	21.52	23.77	23.15	24.99	22.83	22.82	23.57	22.54	24.70
H08	21.52	23.80	23.74	22.38	23.80	23.10	22.09	21.68	23.44	22.32	24.29	24.91
H09	20.87	24.34	23.44	20.48	24.34	23.17	20.62	21.13	23.24	21.19	23.09	23.90
H10	28.09	29.83	28.24	27.31	29.83	28.69	26.78	27.74	28.05	28.14	28.86	28.56
H11	27.68	28.87	29.02	27.21	28.87	27.76	26.82	28.89	28.11	28.13	28.78	28.57
H12	27.96	29.08	28.18	27.46	29.08	29.16	26.52	27.79	26.73	27.91	28.55	28.554

Table S6. Fold regulation and p-Values of genes associated with fatty acid metabolism analyzed in the study. Fold Regulation (comparing to control group)

Position	Gene Symbol	Group 1		Group 2		Group 3	
		**Fold Regulation**	**p-Value**	**Fold Regulation**	**p-Value**	**Fold Regulation**	**p-Value**
A01	**ACAA1**	-2.01	0.048542	-2.8	0.016388	-2.21	0.027894
A02	**ACAA2**	-1.03	0.776242	-1.03	0.792344	1.18	0.714739
A03	**ACAD10**	6.4	0.6315	6.82	0.524766	8.11	0.318698
A04	**ACAD11**	1.5	0.70867	1	0.607066	1.29	0.974287
A05	**ACAD9**	-2.88	0.344697	-3.72	0.322925	-2.93	0.342622
A06	**ACADL**	-12.68	0.369737	1.18	0.416386	1.11	0.417011
A07	**ACADM**	1.04	0.708738	32.85	0.006002	9.59	0.000668
A08	**ACADS**	-1.4	0.251221	-1.7	0.134504	-1.74	0.125277
A09	**ACADSB**	-1.11	0.649403	-1.35	0.168952	-1.5	0.139147
A10	**ACADVL**	-1.51	0.191533	-1.39	0.257033	-1.53	0.043931
A11	**ACAT1**	-1.24	0.5143	-1.33	0.557376	-1.47	0.071616
A12	**ACAT2**	1.02	0.859647	-1.4	0.137779	-1.31	0.524461
B01	**ACOT1**	-8.21	0.373023	-13.43	0.371535	-7.89	0.373397
B02	**ACOT12**	1.16	0.752789	1.66	0.866835	-1.03	0.429374
B03	**ACOT6**	-2.4	0.280458	-3.71	0.201734	-1.86	0.355934
B04	**ACOT7**	1.72	0.035566	-1.43	0.045815	1.05	0.767286
B05	**ACOT8**	-1.96	0.280723	-2.73	0.203106	-1.85	0.2926
B06	**ACOT9**	4.06	0.24632	3.71	0.354409	2.93	0.658994
B07	**ACOX1**	-1	0.99261	-1.22	0.111804	-1.6	0.04958
B08	**ACOX2**	-1.34	0.340069	1.04	0.927867	-1.29	0.561817
B09	**ACOX3**	-1.34	0.169607	-1.94	0.000816	-1.88	0.019001
B10	**ACSBG1**	-1.07	0.796352	1.22	0.574991	1	0.941453
B11	**ACSBG2**	-1.78	0.077905	-5.38	0.003044	-3.58	0.004257
B12	**ACSL1**	-1.48	0.607042	-1.89	0.535272	-1.49	0.552809
C01	**ACSL3**	-3.39	0.132828	-1.63	0.318414	-2.33	0.201804
C02	**ACSL4**	2.89	0.642993	3.05	0.750924	2.51	0.651126
C03	**ACSL5**	-4.49	0.009226	-3.12	0.000959	-1.68	0.035302

Disease Models & Mechanisms • Supplementary information

C04	**ACSL6**	1.22	0.250416	1.1	0.600237	6.3	0.073107
C05	**ACSM3**	-4.38	0.312885	-4.52	0.308799	-3.68	0.325518
C06	**ACSM4**	-7.8	0.359158	-5.88	0.363639	-6.1	0.362462
C07	**ACSM5**	-1.93	0.074261	-3.62	0.015483	-3.07	0.022552
C08	**ALDH2**	-1.43	0.374609	-1.08	0.667734	-1.64	0.252543
C09	**BDH1**	-1.27	0.067749	1.12	0.609952	-1.23	0.609427
C10	**BDH2**	-2.1	0.088246	-1.59	0.339097	-1.39	0.282793
C11	**CPT1A**	-1.45	0.160809	-1.31	0.862862	-1.32	0.160847
C12	**CPT1B**	-1.71	0.08074	-2.98	0.005024	-1.9	0.062988
D01	**CPT1C**	-1.82	0.207438	-1.34	0.403861	-1.61	0.227743
D02	**CPT2**	-3.14	0.168202	-5.11	0.11551	-4.74	0.122737
D03	**CRAT**	5.33	0.288872	4.32	0.550669	6.17	0.172267
D04	**CROT**	5.09	0.292138	5.71	0.248712	7.48	0.053914
D05	**DECR1**	2.98	0.60495	2.62	0.662377	2.33	0.838443
D06	**DECR2**	1.25	0.818553	-2.05	0.213949	-1.02	0.532141
D07	**ECHS1**	1.07	0.550888	-1.16	0.346152	-1.62	0.005287
D08	**ECI2**	-1.14	0.593853	-1.2	0.594086	-1.39	0.152046
D09	**EHHADH**	-1.7	0.162567	1.31	0.572161	-1.03	0.754397
D10	**FABP1**	-12.41	0.116284	-28.9	0.11077	-4.48	0.136404
D11	**FABP2**	-3.32	0.103371	-5.06	0.085761	-3.32	0.11947
D12	**FABP3**	-1.26	0.289409	-1.24	0.975776	1.29	0.390821
E01	**FABP4**	-1.39	0.235217	-1.58	0.773429	-1.76	0.452171
E02	**FABP5**	1.51	0.50594	-2.18	0.301157	-1.74	0.399356
E03	**FABP6**	-1.86	0.250142	-3.53	0.083747	-1.71	0.262328
E04	**FABP7**	-1.5	0.143765	-3.67	0.004842	-2.18	0.009357
E05	**FASN**	1.09	0.758268	-1.7	0.152533	-1.84	0.155805
E06	**GCDH**	-1.12	0.364784	-1.41	0.039111	-1.33	0.223062
E07	**GK**	-1.11	0.792326	1.58	0.368644	-1.3	0.554187
E08	**GK2**	-2.1	0.186509	-3.57	0.09803	-2.22	0.168222
E09	**GPD1**	1.22	0.595872	-1.04	0.519851	-1.84	0.319307
E10	**GPD2**	-1.7	0.131449	-1.48	0.283446	-1.32	0.263873
E11	**HADHA**	-1.28	0.069132	-1.49	0.103464	-1.49	0.027429
E12	**HMGCL**	-1.09	0.373878	-1.13	0.553173	-1.05	0.794041

F01	**HMGCS1**	-1.59	0.112293	-1.94	0.047888	-1.7	0.362526
F02	**HMGCS2**	-3.45	0.230846	-5.37	0.195291	-3.95	0.227631
F03	**LIPE**	-1.69	0.119489	-2.51	0.037891	-1.94	0.078766
F04	**LPL**	1.67	0.369455	1.99	0.029435	2.27	0.094906
F05	**MCEE**	-1.2	0.326859	1.01	0.827637	-1.05	0.828492
F06	**MUT**	-1.39	0.131583	-1.33	0.30076	-1.06	0.801114
F07	**OXCT2**	-1.59	0.369865	-1.15	0.616168	-1.32	0.50045
F08	**PECR**	1.51	0.115709	1.36	0.267124	1.27	0.154291
F09	**PPA1**	-1.13	0.492561	-1.14	0.69016	-1.2	0.252519
F10	**PRKAA1**	-1.06	0.941731	1.13	0.603804	1.06	0.766058
F11	**PRKAA2**	-1.02	0.943831	-1.01	0.805619	-1.21	0.505089
F12	**PRKAB1**	1.26	0.496432	1.35	0.255236	1.24	0.30915
G01	**PRKAB2**	-1.67	0.147342	-1.2	0.524918	1.1	0.82888
G02	**PRKACA**	-1.52	0.238998	1.28	0.522283	1.39	0.318521
G03	**PRKACB**	-1.02	0.887202	-1.05	0.776717	1.38	0.152692
G04	**PRKAG1**	-1.13	0.401189	-1.08	0.846456	1.11	0.465316
G05	**PRKAG2**	-1.09	0.663927	-1.05	0.852306	1.03	0.838065
G06	**PRKAG3**	-1.95	0.373751	-3.1	0.710624	1.84	0.372498
G07	**SLC27A1**	-1.24	0.239276	-1.55	0.105537	-1.8	0.112541
G08	**SLC27A2**	-1.21	0.576657	1.42	0.51309	-1.11	0.692374
G09	**SLC27A3**	-1.05	0.835354	-1.17	0.40721	-1.25	0.387841
G10	**SLC27A4**	1.3	0.17472	1.18	0.21304	-1.38	0.188936
G11	**SLC27A5**	-1.56	0.176814	-3.6	0.008332	-1.99	0.096795
G12	**SLC27A6**	-1.87	0.327645	2.1	0.434841	1.05	0.579324
H01	**ACTB**	1.01	0.988641	-1.15	0.684315	-1.13	0.692316
H02	**B2M**	-1.42	0.292702	-1	0.815027	1.15	0.712705
H03	**GAPDH**	1.01	0.952745	-1.22	0.796888	-1.34	0.41314
H04	**HPRT1**	-1.15	0.67802	-1.17	0.31048	-1.12	0.193351
H05	**RPLP0**	1.61	0.071266	1.65	0.123996	1.49	0.20647

Disease Models & Mechanisms • Supplementary information

Table S7. Genes and primers used in the study.

Gene	Forward (5'-3')	Reverse (5'-3')
**Tp63**	CACCATGTGAGCTCTTCCTATC	AGGTAGCCTCTTACTTCTCCTT
**Cytokeratin K14**	AGTCCCTACTTCAAGACCATTGAG	GGTTCAACTCTGTCTCATACTTGG
**Cytokeratin K13**	TCAAGACACGTCTGGAGCAG	AAGTCAGACAGTGAGGGGTCT
**Beta-Actin**	ACGTTGCTATCCAGGCTGTGCTAT	CTCGGTGAGGATCTTCATGAGGTAGT
**CDH1**	CGATTAAAGGTGGAGAGAGGACTG	AATGAATGGTGGACAGACACAGG
**MUC1**	AGACGTCAGCGTGAGTGATG	GACAGCCAAGGCAATGAGAT
**MUC4**	GGGAAGAAAGGCCCAACTAC	CTATGCTGACGGGTTGGAAT
**IL6**	AAGCCAGAGCTGTGCAGATGAGTA	GCTGCGCAGAATGAGATGAGTTGT
**IL8**	AGACATACTCCAAACCTTTCCACCC	TCCAGACAGAGCTCTCTTCCATCA
**MMP2**	AGAAGGATGGCAAGTACGGCT	AGTGGTGCAGCTGTCATAGGATGT
**CTNNB1** (Beta-Catenin	TCTGAGGACAAGCCACAAGATTACA	TGGGCACCAATATCAAGTCCAA
**LEF1**	AATGAGAGCGAATGTCGTTGC	GCTGTCTTTCTTTCCGTGCTA
**Wnt2**	CTGACCTGATGCAGACGCAAG	AGGAGCCACCTGTAGCTCTCATGTA
**Wnt7b**	GTCAGGGATGTTTGTCCCACTTG	TCTGGTAGGTCCTTGTGCCACTC
**LAMA5**	CACCGCCCGGAAGGC	CAGGTGACTGGACAGGGAAC
**CyclinD1**	TCTACACCGACAACTCCATCCG	TCTGGCATTTTGGAGAGGAAGTG

Disease Models & Mechanisms • Supplementary information
